# Prioritizing management actions for invasive populations using cost, efficacy, demography and expert opinion for 14 plant species world‐wide

**DOI:** 10.1111/1365-2664.12592

**Published:** 2016-02-22

**Authors:** Natalie Z. Kerr, Peter W.J. Baxter, Roberto Salguero‐Gómez, Glenda M. Wardle, Yvonne M. Buckley

**Affiliations:** ^1^ ARC Centre of Excellence for Environmental DecisionsSchool of Biological SciencesThe University of QueenslandSt LuciaQLD4072Australia; ^2^ Department of BiologyTufts UniversityMedfordMassachusetts02155USA; ^3^ Centre for Applications in Natural Resource MathematicsSchool of Mathematics and PhysicsThe University of QueenslandSt LuciaQLD4072Australia; ^4^ Evolutionary Biodemography LaboratoryMax Planck Institute for Demographic ResearchKonrad‐Zuse‐Straße 1RostockDE‐18057Germany; ^5^ Desert Ecology Research GroupSchool of Biological SciencesUniversity of SydneyNSW2006Australia; ^6^ School of Natural SciencesTrinity Centre for Biodiversity Research, ZoologyTrinity College DublinThe University of DublinDublin 2Ireland; ^7^ Present address: Earth, Environmental and Biological Sciences SchoolQueensland University of TechnologyBrisbaneQLD4000Australia

**Keywords:** biological control, economic sensitivity analysis, efficacy, elasticity, invasive plant species, management, marginal cost, matrix population models, population growth rate, weeds

## Abstract

1. Management of invasive populations is typically investigated case‐by‐case. Comparative approaches have been applied to single aspects of management, such as demography, with cost or efficacy rarely incorporated.
2. We present an analysis of the ranks of management actions for 14 species in five countries that extends beyond the use of demography alone to include multiple metrics for ranking management actions, which integrate cost, efficacy and demography (cost‐effectiveness) and managers’ expert opinion of ranks. We use content analysis of manager surveys to assess the multiple criteria managers use to rank management strategies.
3. Analysis of the matrix models for managed populations showed that all management actions led to reductions in population growth rate (λ), with a median 48% reduction in λ across all management units; however, only 66% of the actions led to declining populations (λ < 1).
4. Each management action ranked by cost‐effectiveness and cost had a unique rank; however, elasticity ranks were often tied, providing less discrimination among management actions. Ranking management actions by cost alone aligned well with cost‐effectiveness ranks and demographic elasticity ranks were also well aligned with cost‐effectiveness. In contrast, efficacy ranks were aligned with managers’ ranks and managers identified efficacy and demography as important. 80% of managers identified off‐target effects of management as important, which was not captured using any of the other metrics.
5. *Synthesis and applications*. A multidimensional view of the benefits and costs of management options provides a range of single and integrated metrics. These rankings, and the relationships between them, can be used to assess management actions for invasive plants. The integrated cost‐effectiveness approach goes well ‘beyond demography’ and provides additional information for managers; however, cost‐effectiveness needs to be augmented with information on off‐target effects and social impacts of management in order to provide greater benefits for on‐the‐ground management.

## Abstract 2

A multidimensional view of the benefits and costs of management options provides a range of single and integrated metrics. These rankings, and the relationships between them, can be used to assess management actions for invasive plants. The integrated cost‐effectiveness approach goes well ‘beyond demography’ and provides additional information for managers; however, cost‐effectiveness needs to be augmented with information on off‐target effects and social impacts of management in order to provide greater benefits for on‐the‐ground management.

## Introduction

Management of invasive plant populations is undertaken to mitigate or prevent environmental, societal or economic losses (Simberloff 2003; Buckley 2008). Given the costs of invasion impact and its management, there have been calls for a greater emphasis on cost‐effectiveness and estimation of the likelihood of success of management actions rather than focusing on ecological information alone (Simberloff 2003). While comparative and synthetic work on the ecology (Ramula *et al*. 2008; Catford, Jansson & Nilsson 2009) and impacts of invasive species (Parker *et al*. 1999; Vilà *et al*. 2011; Ricciardi *et al*. 2013) have progressed, management costs and efficacy have not been incorporated into these comparative frameworks. We therefore lack a general understanding of which information to prioritize when seeking management solutions for problematic invaders.

As demographic processes are central to invasions, population models can inform recommendations for invasive species management (Gurevitch *et al*. 2011; Griffith *et al*. 2016). Population matrix models, in particular, lend themselves to comparative analysis due to their standard construction, analytical perturbation analyses and readily interpretable population dynamic metrics (Silvertown *et al*. 1993; Caswell 2001; Salguero‐Gomez *et al*. 2015). These models have been widely used to model invasions and management (e.g. Ramula *et al*. 2008; Dauer, McEvoy & Van Sickle 2012). Perturbation analysis (sensitivity or elasticity) of matrix models reveals the impact on key parameters such as the population growth rate, λ, of small changes to underlying vital rates (e.g. survival, growth, reproduction) or state transitions (de Kroon, van Groenendael & Ehrlèn 2000). Sensitivity and elasticity analyses have been used to guide management by identifying which vital rates or life stage transitions to target in order to achieve the greatest potential impact on population growth rate (Crouse, Crowder & Caswell 1987; Silvertown, Franco & Menges 1996; de Kroon, van Groenendael & Ehrlèn 2000). Management actions can be ranked by the summed elasticities of the vital rates or life stages that a particular management action targets.

Despite improving management decisions (Crouse, Crowder & Caswell 1987), it has long been recognized that elasticities provide only partial information to managers. Limitations of the use of elasticities for informing management include nonlinear responses of population growth rate to underlying parameter changes (de Kroon, van Groenendael & Ehrlèn 2000), biological limits on possible perturbations (Lubben *et al*. 2008), different efficacy of various management actions (Shea *et al*. 2010) and different costs of various management actions (Buhle, Margolis & Ruesink 2005; Baxter *et al*. 2006). Elasticities are not always representative of large changes resulting from management (de Kroon, van Groenendael & Ehrlèn 2000), and nonlinearity in the response of population growth rate to larger changes in vital rates is common (Ramula *et al*. 2008). Managers may not be concerned with the largest potential decrease in population growth rate, as indicated by elasticities, but with the lowest cost for an achievable reduction in population growth rate, the cost‐effectiveness of the management action and/or additional constraints not captured by current analyses. While efficacy has been incorporated into individual species models (Shea *et al*. 2010) and species’ responses to management over time have been modelled (Hansen & Wilson 2006), efficacy and cost have not been incorporated in a multispecies framework to seek generalizations that would be useful in novel management situations.

Although many managers implicitly consider cost within the decision‐making process, there are a number of benefits associated with explicitly accounting for management cost (Buhle, Margolis & Ruesink 2005). Resources for environmental management are limited, so relying on ecological principles alone can produce outcomes that are less efficient, leading to unsuitable allocation of management resources (Baxter *et al*. 2006). The economic perturbation analysis of Baxter *et al*. (2006) integrates the traditional perturbation analysis of matrix models with the relative cost of management actions, explicitly incorporating the management context of decision‐making together with the demography of species of management concern. Here, we extend Baxter *et al*.'s economic perturbation analysis, which uses cost and demography, to incorporate management efficacy, which we define as the reductions in stage transitions achieved in practice by a management action.

We used existing matrix population models from 14 species of invasive herbs, shrubs and trees, together with data on alternative management actions collected from the literature, land managers and/or researchers. Management prioritization is a multidimensional problem. We compared the ranks of management actions according to three single criteria: the *potential* effect of management on the population (elasticity), how much management reduced population growth rate in practice (efficacy) and the cost of different management actions (cost), and two criteria that integrate multiple aspects of management: the reduction in population growth rate, λ, per $ spent on management (cost‐effectiveness) and expert opinion elicited from the managers or decision‐makers (managers). We assessed these metrics using two management objectives: the greatest possible reduction of λ, slowing the population growth rate, and population decline (λ < 1).

We used these data to test hypotheses motivated by three management relevant questions:

- *Do management actions lead to declining populations?* We hypothesized that managers already use management actions that lead to declining (λ < 1) populations and that management actions used by managers would be effective in practice. We therefore hypothesized that costs and potential effects of management (elasticities) would vary between actions, whereas efficacy of management actions would be high.
- *Do individual metrics drive the cost‐effectiveness ranks?* We compared ranks of management actions by cost‐effectiveness with ranks determined by simpler criteria: demographic elasticity, management efficacy and management cost. Since demographic elasticity, management efficacy and cost are all used to determine cost‐effectiveness, all three might be expected to align with cost‐effectiveness. However, if one component of cost‐effectiveness varied more than others, we might expect that component to provide a better proxy. We used rank comparisons to test the hypothesis that cost and elasticity would be better proxies than efficacy for cost‐effectiveness.
- *What do managers use to rank actions?* Managers may use multiple sources of data to implicitly integrate cost, efficacy and demographic considerations. However, they may use only partial information because of data availability; they may weigh some information sources over others; or they may take into account externalities not captured by our cost‐effectiveness approach, such as indirect effects of management (Buckley, Bolker & Rees 2007; Firn, House & Buckley 2010; Buckley & Han 2014). We compared cost‐effectiveness, cost, elasticity and efficacy ranks with ranks elicited from managers to test whether managers’ ranks align with any of these metrics more than others. We also used content analysis of managers’ justifications for their ranks to identify additional management considerations not captured in the cost‐effectiveness analysis.

## Materials and methods

### Demographic Data

We used published matrix population models for invasive plant species with available management information, developed for low‐density populations. Matrix population models were sourced from the literature (including several used in Ramula *et al*. 2008) where we sought all published matrix models for invasive species under management and are currently available in the compadre Plant Matrix Database (Salguero‐Gomez *et al*. 2015). We used a subset of these for which we could find management data. We identified 17 management units from 14 species across five countries from a wide range of environmental contexts including national parks, city parks, scientific reserves and rangelands (see Appendix S1 in Supporting Information which explains the terms used and Appendix S2 which details the demographic information). A management unit is one population with a unique suite of management actions and matrix population models. We used multiple management units for two species, *Carduus nutans* (Appendix S10) and *Cytisus scoparius* (Appendix S13) with substantial differences in demography and management. Management actions were ranked within management units. See Appendix S2 for a list of species and sources and Appendix S3 for a map of locations of management units. The matrix elements of the matrix population models for each management unit were arithmetically averaged across years for a given site and between similar/close sites in order to determine the average population processes applicable when management is applied (see Tables S4.1 and S4.2 for details). Management unit was used as the unit of replication throughout the analyses.

### Management Data

We conducted a search of the grey and peer‐reviewed literature (ISI Web of Science, Google Scholar and Google) for studies that report management cost and efficacy on invasive plant species. We used search terms to find species‐specific management data such as ‘cost’, ‘efficacy’, ‘management’ and both the scientific and common names of the species. When available, we used efficacy values determined from experimental studies.

We collected site‐specific management data from the locations where the matrix population models were developed (see Appendix S2 for original sources and Appendix S4 for management data) by contacting government departments, universities and other agencies affiliated with the study locations, similar sites nearby or within the same region. We elicited information on best management actions for the species in the study area from managers via phone and email surveys using the following questions:

- What control methods are used at the study site or within the region?
- What vital rates or life stages do these control methods target?
- In practice, how effective are these control methods when taking into account both the efficacy of the control methods and the accuracy of control application by managers, contractors and/or volunteers?
- How much do these control methods cost per unit area?

For question (ii), we used the size or developmental characteristics used to construct the stages in the matrix models to help distinguish what life stages, transitions or vital rates are targeted when using different control methods. Once all management data for all actions were collected, we emailed the same managers and asked them to rank all these methods from best to worst according to their expertise and opinion. We also asked managers to provide a brief reason for why they ranked the methods in this order, for example efficacy, cost, off‐target impacts and social impacts.

We excluded biological control as a management action from our study because of the difference in cost structure and temporal scale from local site methods. Biological control typically has a large initial capital cost and low ongoing costs, while the effects are mostly seen in the long term. Out of the 14 species, four had viable biological control options with recorded impacts on populations within the region/country of concern. Another three species have had biocontrol agents released and successfully established, yet no significant impacts on the population were recorded. Local site management actions were therefore the only effective management actions for >70% of the species analysed here, and in all cases, including those where biocontrol is in operation, managers reported using local site management actions.

Costs were normalized by units of measurement (cost per hectare) and currency (USD; 17 February 2012, www.oanda.com), to enable cross‐site and cross‐species comparisons. Cost estimates made before 2011 were converted to the equivalent value in 2011 using inflation rates from local reserve banks.

We used management data from 44 managers (of which 19 managers provided ranks) across 17 management units for 14 species (see S4.1 for summary data on management). Across all management units, there were a total of 55 unique management actions including herbicides, manual (e.g. hand pulling), physical (e.g. bulldozing), site‐based (e.g. prescribed fire, grazing) and combination (e.g. cut stump) methods. As several management actions were applied across multiple management units, there were 82 management action × management unit combinations in total (Appendix S1).

### Analyses

We calculated elasticity, efficacy and cost for each management action and used these metrics to calculate cost‐effectiveness of population growth rate (λ) reduction for each management unit. Cost‐effectiveness was defined as the maximum reduction in λ per USD spent per hectare (from Baxter *et al*. 2006). All notations and definitions are listed in Appendix S5.

We calculated the elasticity of λ, which is the proportional change in population growth rate resulting from small perturbations to matrix elements, for each management action *x* by summing the elasticity values of each life stage transition affected by management, *a*_*ij,x*_ (see Caswell 2001, p. 206–258)

(eqn 1) $$e_{x}=\sum_{i,j}\frac{a_{ij,x}}{\lambda_{x}}\frac{\partial \lambda_{x}}{\partial a_{ij,x}},$$

where matrix element *a*_*ij,x*_ represents the transition from life stage *j* to life stage *i* for the transitions affected by management action *x*.

For efficacy, we determined by how much each transition rate affected by control method *x* was reduced. Matrix element efficacy *f*_*ij,x*_ was defined as the proportional reduction in a matrix element *a*_*ij*_ due to management action *x*. We applied *f*_*ij,x*_ to the relevant transitions and calculated population growth rate for the managed population (λ_*x*_). We defined efficacy of a management action as the percentage change in λ achievable from management:

(eqn 2) $$\Delta \lambda_{x}=\frac{\lambda_{0}-\lambda_{x}}{\lambda_{0}}\times 100,$$

where λ_0_ is the unmanaged population growth rate. Management was assumed to have been applied at the appropriate intensity to achieve the stated efficacy and management has no subsequent indirect effects on matrix elements from estimated changes in transition rates.

We calculated cost as the expense invested in management action *x* per hectare, *c*_*x*_. We then used elasticities, efficacies and costs within the economic sensitivity analysis developed by Baxter *et al*. (2006). We calculated the cost‐effectiveness (marginal efficiency, Baxter *et al*. 2006) of management actions for reducing λ as the reduction in population growth rate per USD ($) per hectare. The marginal cost is a standard economic function defined as the total cost of a unitary change in a parameter (Baxter *et al*. 2006; Mankiw 2012). We calculated the marginal cost (*m*_*ij,x*_) as the cost per hectare of control method *x* (*c*_*x*_) for the estimated or observed change in the demographic transition managed (the product of the managed matrix element (*a*_*ij,x*_) and matrix element management efficacy (*f*_*ij,x*_)):

(eqn 3) $$m_{ij,x}=\frac{C_{x}}{a_{ij,x}f_{ij,x}}$$

The cost‐effectiveness (*g*_*ij,x*_) of managing a matrix element is the sensitivity of the corresponding matrix element (*s*_*ij*_) normalized by its marginal cost (*m*_*ij,x*_) (Baxter *et al*. 2006), which is summed over all matrix elements (*a*_*ij*_) that are influenced by each management action to determine the cost‐effectiveness of a management action (*g*_*x*_):

(eqn 4) $$g_{x}=\sum_{ij}\frac{s_{ij}}{m_{ij,x}}$$

Management of invasive species often has the goal of reducing population size or achieving local eradication. To achieve this goal over the long term, it is necessary that λ < 1. We used two management objectives to compare management actions: (i) actions leading to any reduction in λ and (ii) the subset of actions that can result in λ *<* 1, leading to declining populations through time and, ultimately, a population's local extinction.

We ranked management actions according to their values from each of the five management metrics: elasticity, efficacy, cost, cost‐effectiveness and managers. Actions were ranked from best (1) to worst (*n*), which was low to high for cost and managers and high to low for elasticity, efficacy and cost‐effectiveness. Where ranks were tied (e.g. due to exactly the same elasticities for two actions within a management unit), we calculated the mean rank and assigned this to each of the tied ranks.

In order to determine how individual metrics influence the cost‐effectiveness and managers’ ranks, comparisons were made between cost‐effectiveness and the cost, efficacy, elasticity and managers’ metrics. In addition, we compared managers’ ranks with elasticity, cost and efficacy, resulting in seven comparisons between the five management metrics per management unit. As the number of management actions within each management unit differed and we compared the alignment of ranks between management units, standard rank comparison tests were inappropriate. We developed a rank mismatch index to represent the lack of alignment between two sets of ranks and used this to estimate rank mismatch between two ranking criteria among management units. We calculated the sum of the absolute differences in ranks, *d*, between two management criteria, a measure of matching failure in a sample. We normalized the matching failure by the maximum possible matching failure, *d*_max_, to obtain the rank mismatch index, *h*. For example, the ranks of three management actions (1,2,3) and (3,2,1) have a summed absolute difference in ranks of 4, which is also the maximum achievable, giving a rank mismatch index *h *=* *1.

(eqn 5) $$d=\sum_{n}^{i=1}\left|k_{1,x}-k_{2,x}\right|,$$

where *k* is the rank for management action *x *=* *1 to *n* of *n* management actions, and the ranking criteria being compared are subscripted as 1 and 2.

(eqn 6) $$h=\frac{d}{d_{\max}},$$

where *d*_max_ is calculated as *d* using two samples of ranks which are unique and sequential: *k*_1_ = 1:*n* and *k*_2_ = *n*:1 for *n* management actions.

In order to determine whether the observed rank mismatches were significantly different from random mismatches, we calculated the expected random mismatch for each unique number of management actions in a management unit. The expected random mismatch was calculated as the average rank mismatch over all possible rank permutations compared with the first permutation and varies according to the number of management actions in a management unit:

(eqn 7) $$l=\frac{\sum_{n!}^{j=1}h_{j}}{n!},$$

where *j* is the number of permutations of *n* ranks and *h*_*j*_ is the rank mismatch index for each possible rank permutation. The number of ranks within a management unit ranged from 2 to 13, and the expected random mismatch ranged from 0·5 (two actions) to an asymptote of 0·667 (*l* for 13 management actions was not calculated due to the large number of possible permutations so *l* was calculated from 100 000 random permutations of 13 ranks). Expected random mismatch values for each unique number of management actions were also generated using 100 000 random samples of ranks with replacement to simulate tied ranks. The expected random mismatch values from the resampling procedure with replacement were within 0·02 of *l* calculated numerically or calculated from random permutations (without replacement). One‐sample *t*‐tests for rank alignment were used for each rank comparison by subtracting the expected random match (1−*l*_*r*_) appropriate for the number of management actions from the observed match (1−*l*_*o*_). If the observed match was no different to the expected random match, resulting in values around 0, the null hypothesis would not be rejected. If the observed match was larger than the expected random match, resulting in positive values, this would support an alternative hypothesis of better than random alignment of ranks. Management units were used as the unit of replication in these tests.

Content analysis was used to analyse manager survey responses to identify common decision‐making factors across the species in our study (see S6 for managers’ responses). For the content analysis, we analysed textual answers to our survey questions to design appropriate categories in which all synonymous words and phrases within the text were grouped (Krippendorff 1980). We found five distinct categories: cost, efficacy, demographic considerations, environmental impacts and time consumption. However, all other management considerations that appeared less regularly within responses were documented, including social considerations or method viability across densities, in order to identify management considerations not taken into account in our analyses. Delineating such categories allowed us to determine key decision‐making factors for each individual species and to determine the appropriateness of individual criteria for elicited management concerns. We used the results of the content analysis to interpret differences between manager ranks and cost‐effectiveness ranks.

We used the statistical program r v.3.0.2 (R Core Team 2013) for analyses, models and plotting. Data used can be found in Dryad and in the supporting information (Appendices S2–S4 and S6 provide general data, and Appendices S8–S20 provide species‐specific data). Matrix population models are archived in the compadre Plant Matrix Database ( www.compadre-db.org).

## Results

**Figure 1:**
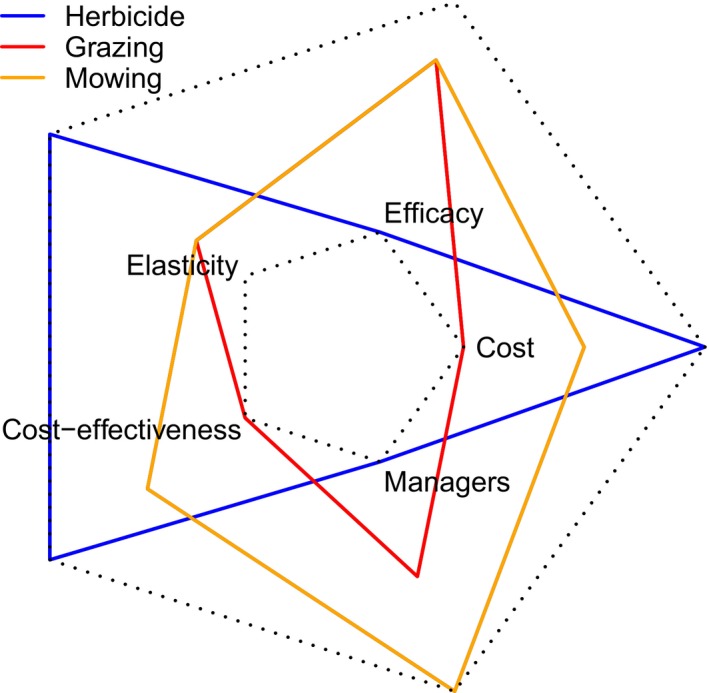
The ranks of management strategies according to each of the ranking criteria: efficacy, cost, managers, cost‐effectiveness and elasticity. Each coloured polygon shows one of the three management actions for the mean matrix (2002–2004) of *Agropyron cristatum* for each of the five ranking criteria on the vertices. The outer dotted pentagon represents the lowest possible ranking of a management strategy on all five criteria (ranked 3), and the inner dotted pentagon shows the highest possible ranking of a management strategy on all five criteria (ranked 1). Smaller polygons have higher ranks. If the criteria were all equally weighted, the best performance overall would be represented by the smallest polygon area; in this case, the red polygon represents grazing. However, it is likely that the criteria would be given unequal weightings depending on stakeholder values.

Figure 1 shows the multidimensional nature of management ranking metrics for a single exemplar species (*Agropyrum cristatum*). Ranks of the management actions differ depending on which metric is used, some management actions perform well under several metrics, and others perform well only under one or two metrics.

### Does Management Lead to Declining Populations?

All management actions led to some reduction in population growth rate (λ); the median reduction in λ due to management was 48%, with a minimum reduction of 4% and up to a maximum reduction of 98%. While reducing λ may be a useful management objective to ameliorate impacts, if the goal of management is to locally eradicate a population, then actions that lead to λ *<* 1 are necessary. The shrub *Lespedeza cuneata* (Schutzenhofer, Valone & Knight 2009) and the tree *Pinus nigra* (Caplat, Nathan & Buckley 2012) were the only species where no management actions were able to achieve a declining population (managed population growth rate always remained >1). Of the remaining species where at least one management action could produce declining populations, we found that 66% of management actions across 12 species (86%) and 14 management units could reduce λ below one.

Across all management units, the actions that achieved a declining λ had the same range of cost and cost‐effectiveness values as the full suite of actions. However, the range of elasticities and efficacies for declining population actions was truncated due to an increase in their minimum values (Table 1). As expected, actions resulting in a declining population had higher median elasticity, efficacy and cost‐effectiveness compared with all other actions. Interestingly, the mean management cost per hectare for the subset of declining population actions was substantially higher (US$819·10 ± 312·77) than the mean cost for all actions (US$660·30 ± 212·54) (see Table 1 for similar trend in median values).

**Table 1:** Summary statistics for each management ranking metric for all actions and for declining population actions for 17 management units of 14 invasive plant species. The % alignment of each management ranking metric with cost‐effectiveness and with managers’ ranks is shown with significant alignment shown in boldface (elasticity and cost are significantly aligned with cost‐effectiveness ranks, and efficacy is significantly aligned with managers’ ranks)

	Median (range)		Percentage alignment (1 – *l*)* *×* *100	
	All actions	Declining population actions	Cost‐effectiveness	Managers’ ranks
Elasticity (potential reduction in λ)	0·53 (0·07–1)	0·63 (0·26–1)	**53**	48
Efficacy (% reduction in λ achieved)	48·2 (3·96–98·49)	61·68 (13·11–98·49)	44	**50**
Cost (USD ha^−1^)	USD$76·62 ha^−1^ (0·01–14 040)	USD$46·22 ha^−1^ (0·01–14 040)	**85**	29
Managers (ranks only)			41	–
Cost‐effectiveness (reduction in λ per USD spent per ha)	8·8 × 10^−3^ USD$^−1^ ha^−1^ (0–327·5)	0·01 USD$^−1^ ha^−1^ (0–327·5)	–	41

### Do Individual Metrics Drive the Cost‐Effectiveness Ranks?

**Figure 2:**
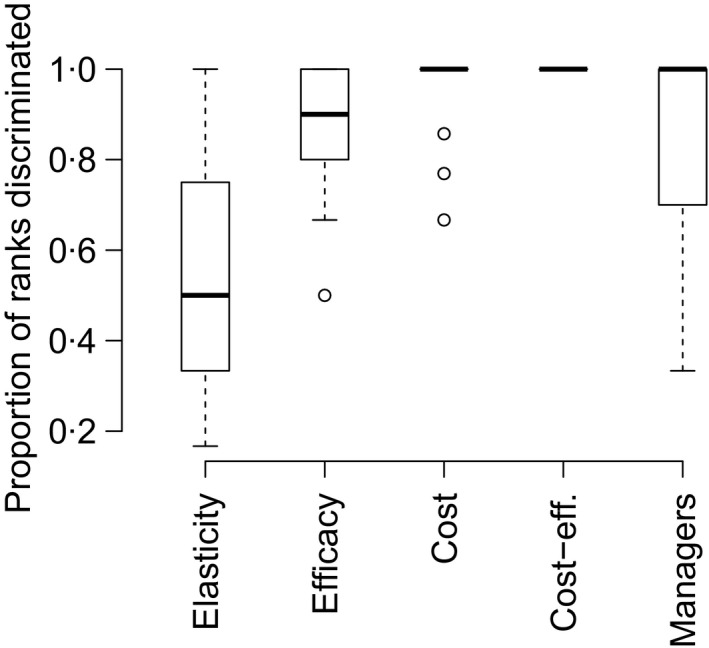
Boxplot of the discrimination ability of each management metric to distinguish between the full suite of actions for each management unit. The *y*‐axis represents the proportion of actions for each management unit that receives a unique rank. If the value is one, then all management actions receive a unique rank according to that criterion. The bold line shows the median proportion, and the box represents the interquartile range with whiskers extending to the 95th and 5th percentiles, and outliers are shown as points. Data are shown for all management actions.

Elasticity ranks provided no discriminatory power between management actions (all actions held the same rank) in 41% of management units and full discrimination between actions (each action receiving a unique rank) in 24% of management units. The only species receiving full discrimination of actions with elasticity analysis (all actions with a unique rank) were those with few management options targeting different stages, for example *Ardisia elliptica* populations with only two actions (Koop & Horvitz 2005), *Rubus armeniacus* with only three actions (Lambrecht‐McDowell & Radosevich 2005) and *Cytisus scoparius* with only two to three actions (Parker 2000; Stokes, Buckley & Sheppard 2006), depending on study site. Efficacy analysis provided more discrimination between management actions than elasticity, with cost, manager and cost‐effectiveness providing similarly high levels of discrimination between actions (Fig. 2).

**Figure 3:**
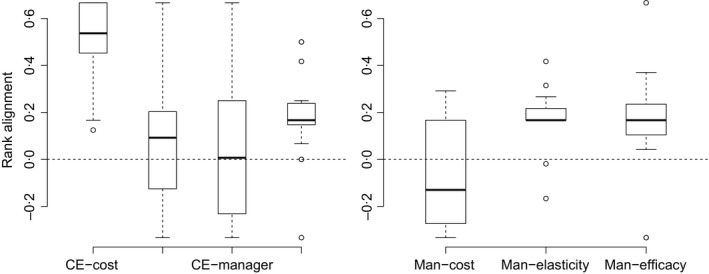
Boxplots showing a) the rank alignment of cost‐effectiveness (CE) ranks for management (Man) actions with ranks determined by cost, efficacy, managers and elasticity and b) the rank alignment of managers’ ranks with ranks determined by cost, elasticity and efficacy. The dashed line at zero indicates rank alignment as expected by chance given the number of management actions per management unit. Values greater than zero indicate higher alignment with cost‐effectiveness than expected by chance. Cost and elasticity ranks of management actions are significantly more aligned with cost‐effectiveness ranks than expected by chance, and efficacy ranks of management actions are significantly more aligned with managers’ ranks than expected by chance. Boxes indicate the interquartile range, whiskers extend to 95th and 5th percentiles, and outliers are shown as points.

Both cost (85%) and elasticity (53%) ranks (Table 1) were significantly better aligned with cost‐effectiveness than expected by chance (cost: *t *=* *10·1, d.f. = 16, *P *<* *0·001, elasticity: *t *=* *3·6, d.f.  = 16, *P *<* *0·005) (Fig. 3a). Management action ranks from efficacy often conflicted with those of the cost‐effectiveness analysis (<45% alignment, Table 1), and one‐sample *t*‐tests showed that rank alignment of efficacy with cost‐effectiveness was no different to that expected by chance (efficacy: *t *=* *1·1, d.f. = 16) (Fig. 3a).

**Figure 4:**
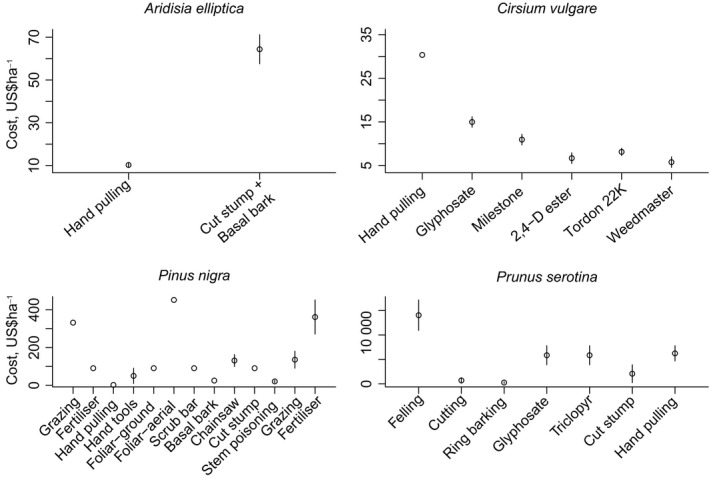
Cost ranges for four species – *Ardisia elliptica, Cirsium vulgare, Pinus nigra* and *Cirsium vulgare* – with available data. Points represent mean cost values, while lines represent cost ranges where available for these species. If cost ranges overlap, that indicates uncertainty in ranks generated using cost and cost‐effectiveness.

We found cost range data for 30·5% out of the 82 management actions across the 17 management units. Overall, we found that only a small subset of actions had overlapping cost ranges for three of the four species, the exception being *Ardisia elliptica* with only two management actions where there was no overlap (Fig. 4).

### How do Managers Rank Actions?

Managers’ ranks of management actions did not align significantly with cost‐effectiveness (*t *=* *0·6, d.f. = 12, Fig. 3b), cost (*t *=* *0·6, d.f. = 10) or elasticity (*t *=* *−1·6, d.f. = 10), but managers’ ranks were significantly aligned with efficacy (*t *=* *3·5, d.f. = 10, *P *<* *0·006; Fig. 3b). The most common factors reported by managers as influencing their management action rankings were efficacy and demographic considerations, which matches well with the alignments shown in Fig. 3b. However, the content analysis showed that 80% of managers considered at least one factor external to the economic sensitivity analysis, such as off‐target environmental impacts and time consumption in the short and long term. Not unexpectedly, there was some inconsistency among managers in their ranking of management actions. For example, for the six species with more than one manager response, the managers differed on the ranks of management actions for *Cytisus scoparius, Lespedeza cuneata* and *Persicaria perfoliata* due to differences in management priorities or prior experience, while managers controlling *Rubus armeniacus*,* Cirsium vulgare* and *Carduus nutans* agreed on the same ranks for management actions.

## Discussion

Multiple criteria relevant to management can be used to rank management actions across species. For the first time, we have carried out a comparative multispecies analysis of cost, efficacy and demography relative to two integrative measures: cost‐effectiveness and managers’ rankings. This approach goes beyond demography, to include other important drivers of management effectiveness. We found that management strategies were ranked very differently depending on the metric used to assess performance. Rankings according to the integrative measure of cost‐effectiveness (a function of demographic sensitivities, efficacy and direct management costs) most closely aligned with cost and demographic rankings, whereas managers’ expert opinion most closely aligned with efficacy. Survey responses from managers also identified efficacy and demography as important drivers of their ranks. However, the vast majority of managers identified a series of additional constraints not taken into account by the metrics presented here, suggesting that cost‐effectiveness needs to be extended to include off‐target environmental, logistical and social considerations.

Overall, our analysis supports the utility of demographic information in determining appropriate management, but also demonstrates that it is necessary to go beyond demography and explicitly consider economic, management efficacy and other considerations when assessing management options. Here, we will concentrate on synthesizing the results of our analysis in order to build broad recommendations for the management of invasive plant species, particularly when only partial information is available. See Appendices S4 and S7–20 for more details on species‐specific management recommendations.

While all management actions slowed population growth rate, only 66% of actions across all 17 management units were able to achieve long‐term declining populations (λ < 1). The range of management options available was more costly if the objective was to achieve declining populations, so it is crucial that recommendations account for management objectives. In rapidly growing populations, there are situations where slowing rather than reversing population growth rate may lead to direct benefits and/or delays in impact. However, it is important to be clear about what the expected benefits are in order to extend the cost‐effectiveness analysis provided here and determine the reductions in impact per $ spent, not just the reduction in population growth rate. Extending this approach to impact would require an understanding of how population abundance changes with population growth rate and how impact scales with abundance (Yokomizo *et al*. 2009). In situations with multiple invasive species and limited resources, cost‐effectiveness could be combined with data on impact to prioritize management among weed species within a region. Impact will have different metrics and directions depending on the system affected, resulting in several impact ‘currencies’ which need to be combined or traded off against each other (Barney *et al*. 2013; Grechi *et al*. 2014).

Based on demographic information alone, we found that several management actions received exactly the same elasticity value, making it difficult to discriminate among management actions, for example for *Cirsium vulgare* where all management actions received the same elasticity values (Appendix S12). The life stage partitioning for the construction of matrix models may limit the differentiation of actions by elasticity analysis. For example, all 13 actions for controlling *Pinus nigra* in New Zealand received one of four elasticity values, where the four‐stage partitioning of its life history used to develop the matrix model was likely to restrict the differentiation of these methods (Caplat, Nathan & Buckley 2012). However, the matrix dimensionality does not have to limit the discriminatory power of elasticities, greater discrimination among elasticity ranks could be achieved by determining how management affects the parameters underlying the transition, and elasticities for the affected underlying vital rates could be summed instead of summing the elasticities for whole affected transitions (limited by the matrix dimension). There is currently a lack of data on the mode of demographic action of management techniques.

Life‐history complexity is also likely to be a determinant of the demographic targets of management and their differentiation by elasticity analysis. Several of the management actions for a single species affected the same transition, and for small matrices (low dimensionality), opportunities for differentiation of management actions in terms of the elasticities of the underlying demographic matrix were limited. For example, management actions for less complex functional forms are likely to be non‐specific in the life stages they target, for example herbicide on herbaceous weeds such as *Cirsium vulgare* (Appendix S12) and *Lespedeza cuneata* (Appendix S15), compared to the more complex life cycle of shrubs and trees where different management actions might target a larger range of stages and vital rates, for example hand pulling of seedlings vs. chainsawing of mature trees such as for *Pinus nigra* (Appendix S18).

Elasticity analysis as used here involves the assumption that negative effects of management on one set of vital rates are not compensated for with positive responses in other vital rates, for example via a density dependence mechanism. Use of integrative elasticities to account for covariation among vital rates would help to resolve this, but this requires detailed knowledge of how management affects vital rates and multiple transitions (Van Tienderen & Van Hinsberg 1996; van Tienderen 2000). Management experiments should be used not just to assess the overall effectiveness of management, but also to determine the positive and negative effects of management on underlying demographic rates.

Matrix models for invasive species under active management are scarce, and many managers do not have the available resources to build these ‘data‐heavy’ models. Therefore, finding simpler proxy rankings for management actions would be invaluable under many management situations. For management situations with no financial restraints, efficacy analysis alone could be used for decision‐making assuming that the management objective is to reduce population growth rate as much as possible. In fact, efficacy was the only management metric that was significantly aligned with managers’ ranks, indicating that it may already play this role. Efficacy provided greater discrimination than elasticity and varied across management actions, yet efficacy still lacked the ability to discriminate between actions for several cases (Fig. 2). Perhaps, this was due to uncertainty regarding the effects of management on target transitions rates, entailing reliance on coarser estimates of efficacy.

Efficacy was not well aligned with cost‐effectiveness for many management units, suggesting that efficacy was not a major determinant of cost‐effectiveness. However, the management actions we found data for were already at the upper end of the efficacy spectrum, since data on ineffective management were rarely reported. All management actions resulted in a reduction in population growth rate of at least 4% with a mean reduction of nearly 50% and some extremely high efficacies (>98%) were also reported (Table 1).

The ranks of management actions by cost‐effectiveness were most closely aligned with actions ranked by cost alone, followed by a lower but still significant alignment with elasticity. The cost of management is therefore the strongest determinant of the outcome of the economic sensitivity analysis (Fig. 3a). The extremely high discriminatory ability of cost and the relatively low discriminatory ability of elasticity (many tied ranks, Fig. 2) is likely one of the reasons for the lower alignment of elasticity with cost‐effectiveness. Therefore, demographic information may be more useful for species with a more complex life history, where there was a wider range of available management actions that could be targeted at more specific life stages. Cost likely had high discriminatory ability due to the large range in values ($USD 0–14 040 per ha). Since cost seems to be driving cost‐effectiveness, ignoring uncertainty in these cost estimates can lead to misinformed management according to both cost and cost‐effectiveness metrics. For four species with cost ranges, cost uncertainty had low to moderate effects on the ranking of management actions within a management unit (Fig. 4); however, the strong alignment between cost and cost‐effectiveness ranks means that variations in cost will lead to corresponding responses in cost‐effectiveness. Incorporating uncertainty in cost is also unlikely to make alignment between cost‐effectiveness, cost and managers’ ranks stronger. Therefore, our findings of alignment between cost and cost‐effectiveness and lack of alignment between managers’ ranks and both cost and cost‐effectiveness are likely to be robust to the reported cost uncertainty.

A population's demographic rates and associated management costs are unlikely to be fixed (Ehrlén *et al*. 2016) or accurately known, leading to high uncertainty around management decisions. Methods exist to address uncertainty in matrix models that arises through demographic parameters (Deines *et al*. 2007) and in management costs (Salomon *et al*. 2013). These methods typically use alternative approaches to management decisions: at what level of parameter or cost uncertainty would we expect to change our highest ranked strategy? Value of information methods can be used to identify the parameters for which reduced uncertainty will best aid decision‐makers, even if multiple management objectives exist (Runge, Converse & Lyons 2011). In addition, combinations of management strategies may offer some resilience to uncertainty while lowering expected performance (Salomon *et al*. 2013). The methods developed here can be used to assess how management affects alternative demographic metrics such as short‐term transient dynamics which can play a large role in perturbed and variable environments (McDonald *et al*. 2016).

**Figure 5:**
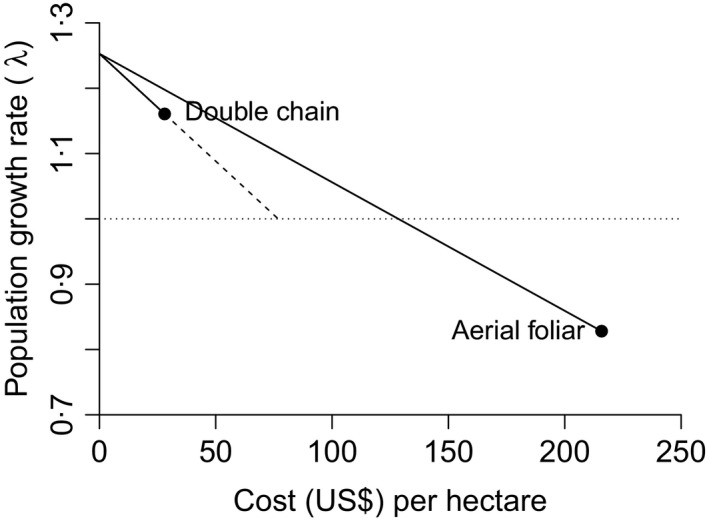
The assumed linear relationship between population growth rate and cost of the two most cost‐effective methods used to control *Parkinsonia aculeata* populations in Australia. Double chain is the most cost‐effective, yet unable to achieve a declining population (λ > 1), while aerial foliar application is the second most cost‐effective, yet able to achieve a declining population (λ < 1). The solid line represents the total reduction in population growth rate resulting from management, while the dotted line represents the additional cost required to achieve a declining population assuming a linear relationship.

The achievable impact of a management action depends on logistical constraints such as the intensity of management that can be applied as well as the potentially nonlinear response of the transition or vital rate targeted (de Kroon, van Groenendael & Ehrlèn 2000). Elasticity analysis assesses the effect on population growth rate of small, linear changes to vital rates that may not be representative of large changes resulting from management carried out *in situ*. Another important management constraint of the economic sensitivity analysis is its inability to predict the shape of the relationship between cost and population growth rate; we refer to this as the cost–λ curve. For example, it may be possible to invest more money on double chain pulling for controlling *Parkinsonia aculeata* to reduce λ below 1 (by increasing efficacy for example, the dashed line in Fig. 5) or, alternatively, save limited resources by spending less money on aerial foliar application, so that λ is only slightly below 1 (Fig. 5; see Appendix S16 for more details). Extrapolating the cost–λ curve for both of these actions assumes that the relationship is linear when, in fact, spending more or less money may not achieve a linear response in population growth rate. Capital costs may intervene with the cost–λ curve, but also this curve assumes that increased costs would result in a greater reduction in number of individuals within the life stages targeted. The solution is to gain a deeper understanding of how demography, and ultimately population impact, varies with the management effort and cost. This either requires empirical management experiments and/or careful modelling of the demographic and wider ecosystem effects of management.

The availability of management actions expands to suit different management contexts as well as to integrate goals other than mitigating the impact of weeds (Pearson & Ortega 2009). Cost‐effectiveness ranks might align better with managers’ ranks in systems with straightforward management objectives, for example maximization of yield in agricultural systems, than in a system that often incorporates indirect and/or difficult to value costs and benefits, for example prevention or reversal of impacts of invaders on biodiversity in nature reserves and parks. Cost‐effectiveness tools alone may be more appropriate to use in conventional cropping systems where the emphasis is on management cost and reduction in population growth rate. Our quantitative cost‐effectiveness analyses did not take into account some of the common externalities associated with management such as off‐target effects on desired natural capital and the services that biodiversity provides (Buckley & Han 2014) or the social and economic gains from provision of grazing or volunteer work.

Content analysis of the manager survey responses indicated that the management decision‐making process incorporated a range of factors not captured in the cost‐effectiveness analysis. While demography and efficacy were often considered when justifying management rankings, we found that off‐target environmental impacts were of equal concern. Even though the cost‐effectiveness analysis is able to incorporate what are perceived as important decision‐making factors, we found that the decision‐making process is usually subject to several additional constraints.

We conclude that a multidimensional view of management options provides a range of single and integrated metrics, and the relationships between them, which can be used to rank management actions for invasive plants. Cost‐effectiveness can be a very useful tool when making management decisions in straightforward systems; it has excellent discriminatory power between management actions and can be used to compare management actions using a single currency (reduction in population growth rate per $ per hectare). We have shown that cost and/or demography can provide useful proxies for cost‐effectiveness. Finally, we show that managers consider a greater range of management constraints than captured in cost‐effectiveness analysis. We have demonstrated that these constraints can be elicited from managers and that efficacy, demography and off‐target effects are considered of primary importance for managers’ rankings. While the comparative cost‐effectiveness approach goes well ‘beyond demography’ and provides additional information for managers, it needs to be augmented with information on off‐target effects and social impacts of management in order to be useful for on‐the‐ground management.

## Author contributions

NZK collected data, analysed data and wrote manuscript. PWJB involved in research design and edited manuscript. RSG and GW edited manuscript. YMB involved in research design, analysed data and wrote manuscript.

## Data accessibility

Demographic data were sourced from the compadre Plant Matrix Database http://www.compadre-db.org/ and are publicly accessible. Management data are available from Dryad Digital Repository doi: 10.5061/dryad.r87d6 (Kerr *et al*. 2016). Additional data and analyses are available as online Supporting Information.

## Supporting information


**Appendix S1.** Hierarchical structure of management data.

**Appendix S2.** Demographic information for the 14 species used.

**Appendix S3.** Continental maps of the population locations.

**Appendix S4.** Management data.

**Appendix S5.** Table of symbols and definitions of model parameters and management objectives.

**Appendix S6.** Responses of survey analysis.

**Appendix S7.** *Agropyron cristatum*.

**Appendix S8.** *Alliaria petiolata*.

**Appendix S9.** *Ardisia elliptica*.

**Appendix S10.** *Carduus nutans*.

**Appendix S11.** *Centaurea stoebe*.

**Appendix S12.** *Cirsium vulgare*.

**Appendix S13.** *Cytisus scoparius*.

**Appendix S14.** *Dipsacus sylvestris*.

**Appendix S15.** *Lespedeza cuneata*.

**Appendix S16.** *Parkinsonia aculeata*.

**Appendix S17.** *Persicaria perfoliata*.

**Appendix S18.** *Pinus nigra*.

**Appendix S19.** *Prunus serotina*.

**Appendix S20.** *Rubus armeniacus*.
