# Supplementary material for: Prioritizing management actions for invasive populations using cost, efficacy, demography and expert opinion for 14 plant species world‐wide
Source: J Appl Ecol. 2016 Feb 22;53(2):305–16. doi: 10.1111/1365-2664.12592 (PMC4949517; doi:10.1111/1365-2664.12592)
Supplement: Supplementary file 1 — Appendix S1. Hierarchical structure of management data. [file JPE-53-305-s001.docx]

**Appendix S1. Hierarchical structure of management data.**

Management data were collected for 17 management units in 14 species, the structure of data for species (*i*), management units (*j*), and management actions (*k*) is shown here. The sample size (*N*) is shown for each level in the data hierarchy, and the ranking of management actions is shown within management units. Most species had just one management unit but *Carduus nutans*  and *Cytisus scoparius* had two and three management units respectively. The number of management actions ranges from 2 to 13 for each management unit. See S4.1 for details.

**
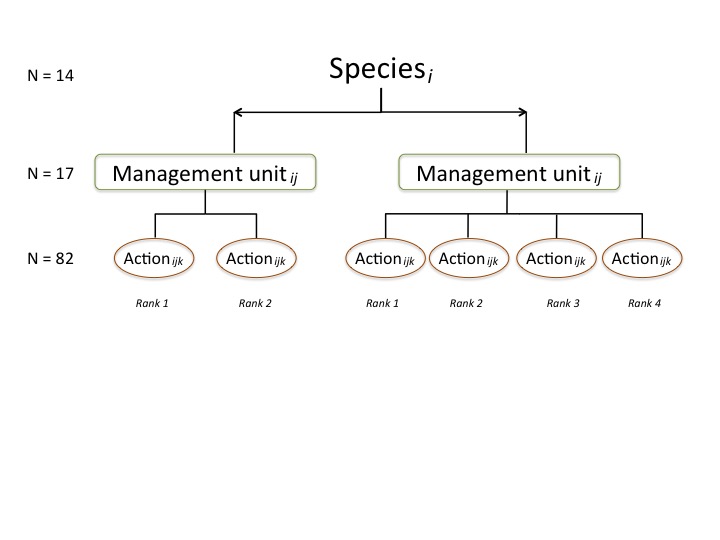
**
