# Supplementary material for: Prioritizing management actions for invasive populations using cost, efficacy, demography and expert opinion for 14 plant species world‐wide
Source: J Appl Ecol. 2016 Feb 22;53(2):305–16. doi: 10.1111/1365-2664.12592 (PMC4949517; doi:10.1111/1365-2664.12592)
Supplement: Supplementary file 2 — Appendix S2. Demographic information for the 14 species used. [file JPE-53-305-s002.docx]

Appendix S2. Demographic information for the 14 species used. Native range range, introduced range, the study location from where the demographic data for the matrix was collected, matrix dimensions, and matrix sources.

| **Species** | **Life form** | **Native range** | **Invasive range** | **Study location** | **No. of published matrices** | **Matrix dim.** | **Matrix Source** |
| --- | --- | --- | --- | --- | --- | --- | --- |
| *Agropyron cristatum*^A^ | Grass | Asia, Europe | North America | Grasslands National Park, Canada | 3 | 8 | (Hansen & Wilson 2006; Hansen 2007) |
| *Alliaria petiolata^K^* | Herb | Asia, Europe | North America | Second growth hardwood forests located on Ridges Land Laboratory, Ohio | 1 | 3 | (Davis *et al.* 2006) |
| *Ardisia elliptica*^D^ | Shrub | Asia | North America, Africa Central America, Oceania | Everglades National Park, Florida | 13 | 8 | (Koop & Horvitz 2005) |
| *Carduus nutans*^B^ | Herb | Asia, Africa, Europe | Asia, Europe, North America, South America, Oceania | New South Wales, Australia; North Island, New Zealand | 2 | 4 | (Shea *et al.* 2010) |
| *Centaurea stoebe*^CD^ | Herb | Europe | Asia, North America, Europe, Oceania | Forested areas in Fort Custer Training Center near Augusta, Michigan, USA | 1 | 5 | (Emery & Gross 2005) |
| *Cirsium vulgare*^B^ | Herb | Asia, Africa, Europe | Asia, Europe, North America, South America, Oceania, Africa | Eastern Nebraska, USA | 1 | 4 | (Tenhumberg *et al.* 2008) |
| *Cytisus scoparius*^EF^ | Shrub | Europe | North America, South America, Oceania, Asia, Africa | City parks and prairie fields of Washington, USA; along the Shoalhaven River, New South Wales, Australia | 9 | 7 | (Parker 2000; Stokes, Buckley & Sheppard 2006) |
| *Dipsacus sylvestris* | Herb | Europe, Asia, Africa | North America, Oceania | Kalamazoo County in Michigan, USA | 7 | 6 | (Caswell 2001) |
| *Lespedeza cuneata*^G^ | Herb | Asia | North America | Oak-Hickory forests and open fields of Missouri, USA | 1 | 6 | (Schutzenhofer, Valone & Knight 2009) |
| *Parkinsonia aculeata*^D^ | Shrub | Americas | Oceania, Africa, Asia, South America | Northern Territory and Western Australia, Australia | 10 | 8 | (Pichancourt & van Klinken 2012) |
| *Persicaria perfoliata* | Herb | Asia | North America | Nature reserves in Pennsylvania, USA | 12 | Varies | (Hyatt & Araki 2006) |
| *Pinus nigra*^I^ | Tree | Europe | Oceania | Mt Barker, New Zealand | 1 | 8 | (Caplat, Nathan & Buckley 2012) |
| *Prunus serotina*^D^ | Tree | North America | Europe | Compiegne forest located in Northern France | 1 | 11 | (Sebert-Cuvillier *et al.* 2007) |
| *Rubus armeniacus*^J^ | Shrub | Asia | North America | Forested areas of Oregon, USA | 1 | 4 | (Lambrecht-McDowell & Radosevich 2005) |

References for native range and known invasive range for these 14 invasive species: ^A^(Rogler & Lorenz 1983), ^B^(Holm *et al.* 1997), ^C^(Emery & Gross 2005), ^D^(Risk 2015), ^E^(Downey & Smith 2000), ^F^(Potter *et al.* 2009), ^G^(Schutzenhofer, Valone & Knight 2009), ^I^(Richardson & Rejmanek 2004), ^J^(Lambrecht-McDowell & Radosevich 2005), ^K^(Welk, Schubert & Hoffmann 2002).

**References**

Caplat, P., Nathan, R. & Buckley, Y.M. (2012) Seed terminal velocity, wind turbulence, and demography drive the spread of an invasive tree in an analytical model. *Ecology,* **93,** 368-377.

Caswell, H. (2001) *Matrix population models: construction, analysis and interpretation,* 2nd edn. Sinauer Associates, Inc., Sunderland, Massachusetts.

Davis, A.S., Landis, D.A., Nuzzo, V., Blossey, B., Gerber, E. & Hinz, H.L. (2006) Demographic models inform selection of biocontrol agents for garlic mustard (Alliaria petiolata). *Ecological Applications,* **16,** 2399-2410.

Downey, P.O. & Smith, J.M.B. (2000) Demography of the invasive shrub Scotch broom (*Cytisus scoparius*) at Barrington Tops, NSW: insights for management. *Austral Ecology,* **25,** 477-485.

Emery, S. & Gross, K. (2005) Effects of timing of prescribed fire on the demography of an invasive plant, spotted knapweed Centaurea maculosa. *Journal of Applied Ecology,* **42,** 60-69.

Hansen, M. & Wilson, S. (2006) Is management of an invasive grass Agropyron cristatum contingent on environmental variation? *Journal of Applied Ecology,* **43,** 269-280.

Hansen, M.J. (2007) Evaluating management strategies and recovery of an invasive grass (Agropyron cristatum) using matrix population models. *Biological Conservation,* **140,** 91-99.

Holm, L., Doll, J., Holm, E., Pancho, J. & Herberger, J. (1997) *World weeds: Natural histories and distribution*. John Wiley & Sons Inc., Canada.

Hyatt, L. & Araki, S. (2006) Comparative population dynamics of an invading species in its native and novel ranges. *Biological Invasions,* **8,** 261-275.

Koop, A. & Horvitz, C. (2005) Projection matrix analysis of the demography of an invasive, nonnative shrub (Ardisia elliptica). *Ecology,* **86,** 2661-2672.

Lambrecht-McDowell, S. & Radosevich, S. (2005) Population demographics and trade-offs to reproduction of an invasive and noninvasive species of Rubus. *Biological Invasions,* **7,** 281-295.

Parker, I.M. (2000) Invasion dynamics of *Cytisus scoparius*: A matrix model approach. *Ecological Applications,* **10,** 726-743.

Pichancourt, J.-B. & van Klinken, R.D. (2012) Phenotypic plasticity influences the size, shape and dynamics of the geographic distribution of an invasive plant. *PLoS ONE,* **7,** e32323.

Potter, K.J.B., Kriticos, D.J., Watt, M.S. & Leriche, A. (2009) The current and future potential distribution ofCytisus scoparius: a weed of pastoral systems, natural ecosystems and plantation forestry. *Weed Research,* **49,** 271-282.

Richardson, D.M. & Rejmanek, M. (2004) Conifers as invasive aliens: a global survey and predictive framework. *Diversity and Distributions,* **10,** 321-331.

Risk, P.I.E.a. (2015) *Aridisia elliptica*.

Rogler, G.A. & Lorenz, R.J. (1983) Crested wheatgrass - early history in the United States. *Journal of Range Management,* **36,** 91-93.

Schutzenhofer, M.R., Valone, T.J. & Knight, T.M. (2009) Herbivory and population dynamics of invasive and native Lespedeza. *Oecologia,* **161,** 57-66.

Sebert-Cuvillier, E., Paccaut, F., Chabrerie, O., Endels, P., Goubet, O. & Decocq, G. (2007) Local population dynamics of an invasive tree species with a complex life-history cycle: A stochastic matrix model. *Ecological modelling,* **201,** 127-143.

Shea, K., Jongejans, E., Skarpaas, O., Kelly, D. & Sheppard, A.W. (2010) Optimal management strategies to control local population growth or population spread may not be the same. *Ecological Applications,* **20,** 1148-1161.

Stokes, K.E., Buckley, Y.M. & Sheppard, A.W. (2006) A modelling approach to estimate the effect of exotic pollinators on exotic weed population dynamics: bumblebees and broom in Australia, *Diversity & Distributions,* **12,** 593-600.

Tenhumberg, B., Louda, S., Eckberg, J. & Takahashi, M. (2008) Monte Carlo analysis of parameter uncertainty in matrix models for the weed Cirsium vulgare. *Journal of Applied Ecology,* **45,** 438-447.

Welk, E., Schubert, K. & Hoffmann, M.H. (2002) Present and potential distribution of invasive garlic mustard (Alliaria petiolata) in North America. *Diversity and Distributions,* **8,** 219-233.
