# Supplementary material for: Prioritizing management actions for invasive populations using cost, efficacy, demography and expert opinion for 14 plant species world‐wide
Source: J Appl Ecol. 2016 Feb 22;53(2):305–16. doi: 10.1111/1365-2664.12592 (PMC4949517; doi:10.1111/1365-2664.12592)

**Appendix S3.** **Continental maps of the population locations** used for the demographic studies (see S2), and where management data were collected for our analyses.


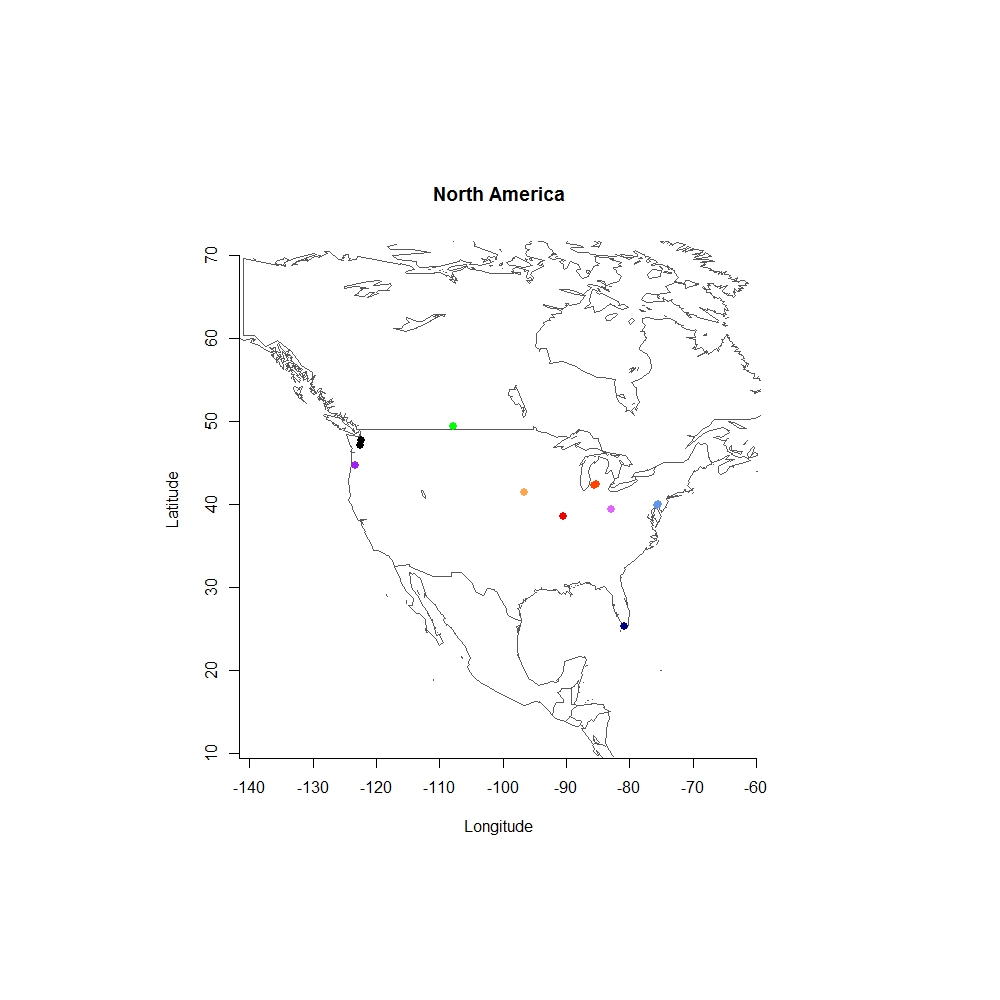

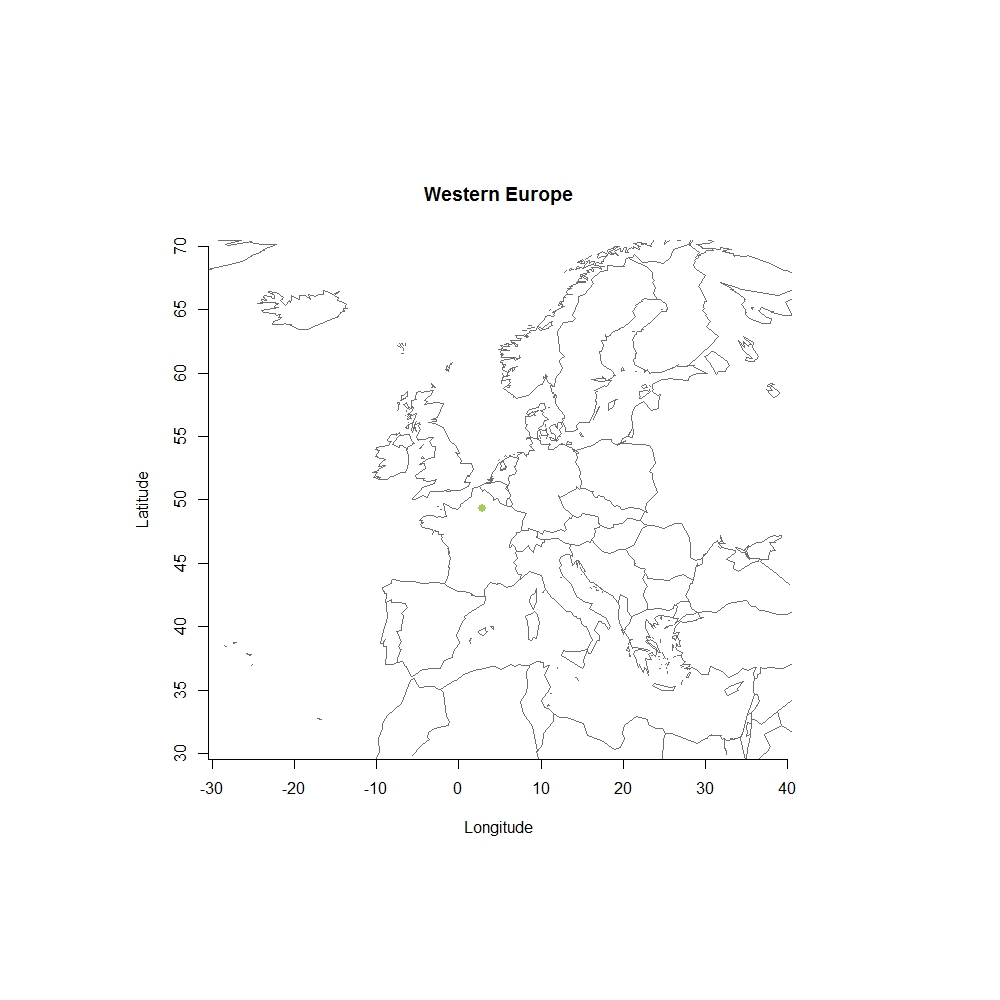


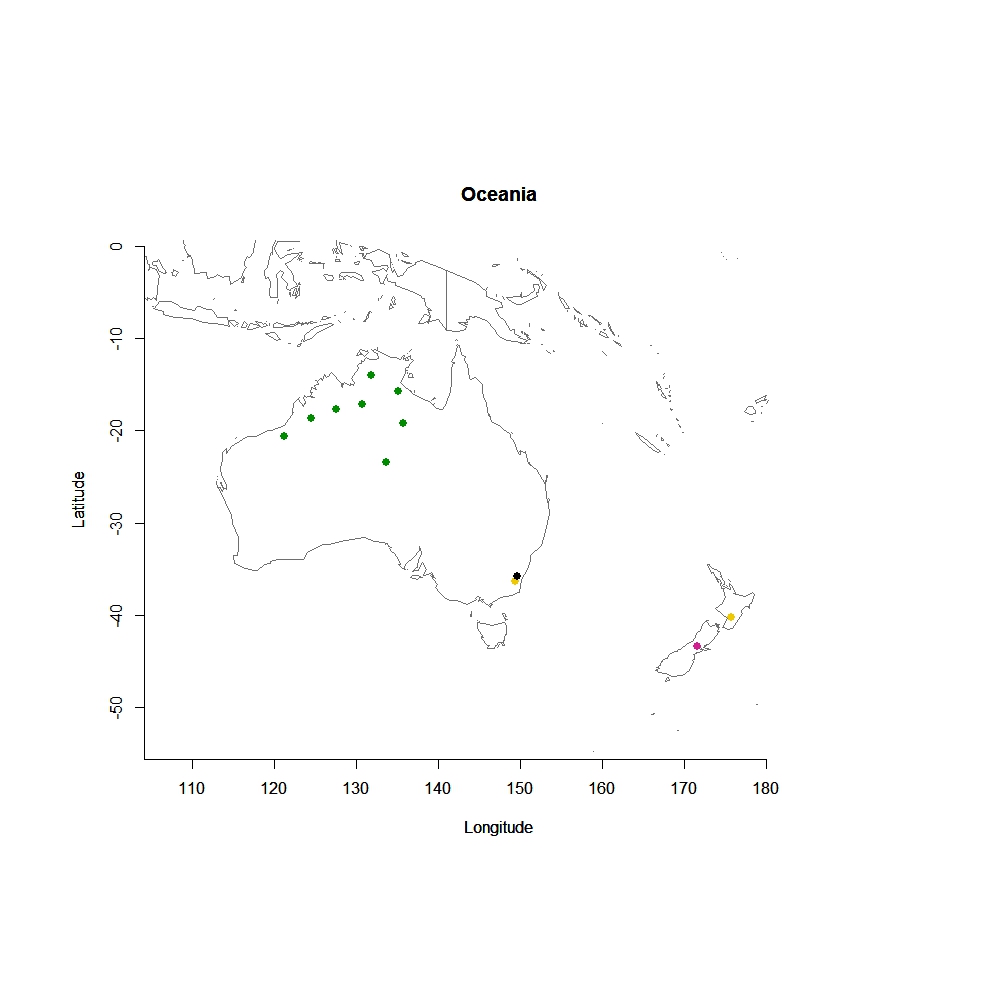

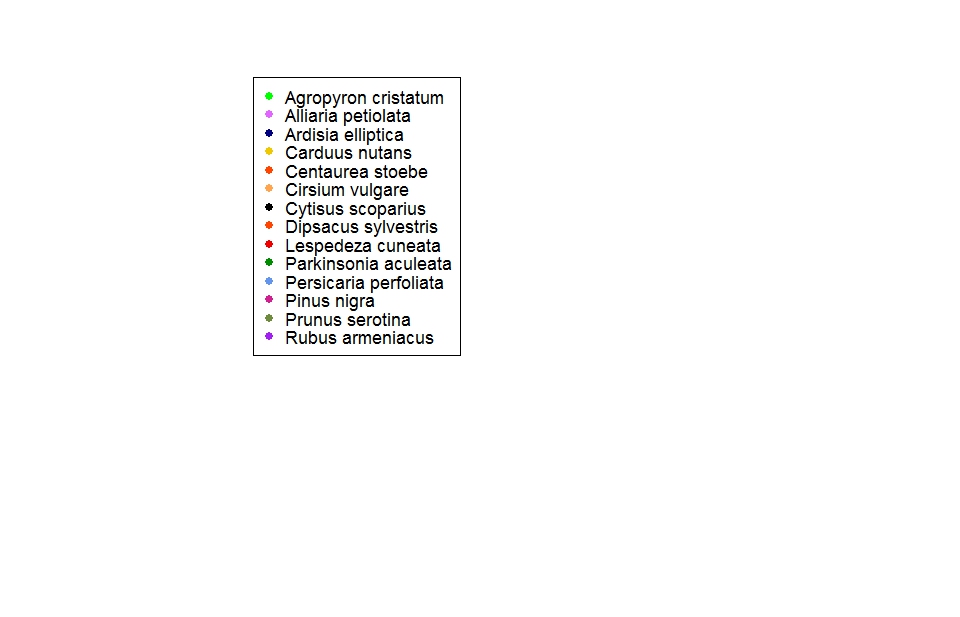

Supplement: Supplementary file 3 — Appendix S3. Continental maps of the population locations. [file JPE-53-305-s003.docx]
