# Supplementary material for: Prioritizing management actions for invasive populations using cost, efficacy, demography and expert opinion for 14 plant species world‐wide
Source: J Appl Ecol. 2016 Feb 22;53(2):305–16. doi: 10.1111/1365-2664.12592 (PMC4949517; doi:10.1111/1365-2664.12592)
Supplement: Supplementary file 4 — Appendix S4. Management data. [file JPE-53-305-s004.docx]

**Appendix S4 Management Data (Tables S4.1 & S4.2)**

**Table S4.1.** List of species with the number of management units (unique suite of actions or different management data for population or species), population sites and transitions across years, management actions (unique method for species or population), matrix source (see S2 for more demographic information), and number of managers that provided management ranks and management data.

| Species | No. of Management Units (MU) | Sites/ transitions | No. of management actions | Matrix source | No. managers providing ranks/data |
| --- | --- | --- | --- | --- | --- |
| *Agropyron cristatum* | 1 | 1/3 | 3 | Hansen and Wilson 2006, Hansen 2007 | 1/3 |
| *Alliaria petiolata* | 1 | 1/1 | 4 | Davis et al. 2006 | 0/0 |
| *Ardisia elliptica* | 1 | 4/3  1/1 | 2 | Koop and Horvitz 2005 | 0/1 |
| *Carduus nutans* | 2 | 1/1 (MU1)  1/1 (MU2) | 3 | Shea et al 2010 | 4/7 |
| *Centaurea stoebe* | 1 |  | 5 | Emery and Gross 2005 | 0/2 |
| *Cirsium vulgare* | 1 |  | 6 | Tenhumberg et al. 2008 | 2/3 |
| *Cytisus scoparius* | 3 | 2/1, 2/2 (MU1)  2/1, 2/2 (MU2)  1/1 (MU3) | 3  3  3 | Parker 2000, Stokes et al. 2006 | 2/7 |
| *Dipsacus sylvestris* | 1 | 7/1 | 2 | Schutzenhofer et al. 2009 | 0/2 |
| *Lespedeza cuneata* | 1 | 1/1 | 6 | Jacquemyn et al. 2005 | 2/2 |
| *Parkinsonia aculeata* | 1 | 8/1 | 10 | Pichancourt and van Klinken 2012 | 1/4 |
| *Persicaria perfoliata* | 1 | 1/2  1/2  1/2 | 6 | Hyatt and Araki 2006 | 2/4 |
| *Pinus nigra* subspp. *laricio* | 1 | 1/1 | 13 | Caplat et al. 2011 | 1/4 |
| *Prunus serotina* | 1 | 1/1 | 7 | Sebert-Cuvillier et al. 2007 | 1/1 |
| *Rubus armeniacus* | 1 | 1/1 | 3 | Lambrecht-McDowell and Radosevich 2005 | 3/4 |

Table S4.2. Management data for the 14 invasive plant species when managed at their study locations between 2011 and 2012. The management actions used at the sites (Table 1), transition rates affected (a_ij_ meaning the transition from life stage j to life stage i), efficacy (%) or managed population model (**B**), cost (US$) per hectare, summed elasticity (*∑e_ij_*_,x_), summed cost-efficiency (∑*g_ijx_*) and the source of management data for each matrix is outlined below. The specific transition rates of each matrix and management efficacy for all methods for *Agropyron cristatum* and prescribed fire to manage *Centaurea stoebe* were obtained from matrices in their corresponding journal articles (see Matrix source, Table 4.1).

| **Species** | **Management strategy (x)** | **Transition/vital rates directly affected by action, *x*** | **Efficacy**  **(% reduction, *f_ij,x_*)** | | **Managed λ*_x_*** | | **Elasticity (*e*_x_)** | | **Efficacy, %**  **(Δλ*_x_*)** | | **US$ per ha**  **(point; range)** | **Cost-effectiveness (*g_x_*)** | |
| --- | --- | --- | --- | --- | --- | --- | --- | --- | --- | --- | --- | --- | --- |
| *Agropyron cristatum* | Grasslands National Park, Canada (λ_0_ = 4.67) | | | | | | | | | | | | |
|  | Grazing | a_5,4_, a_1,5_, a_4,5_, a_6,5_, a_7,5_, a_1,6_, a_4,6_, a_6,6_, a_7,6_, a_8,6_, a_1,7_, a_4,7_, a_8,7_, a_1,8_, a_4,8_, a_8,8_ | See matrix source | 0.898 | | 0.921 | | 80.76 | | 0.01 | | | 327.46 |
|  | Mowing | a_5,4_, a_1,5_, a_4,5_, a_6,5_, a_7,5_, a_1,6_, a_4,6_, a_6,6_, a_7,6_, a_8,6_, a_1,7_, a_4,7_, a_8,7_, a_1,8_, a_4,8_, a_8,8_ | See matrix source | 0.898 | | 0.921 | | 80.76 | | 2.63 | | | 1.245 |
|  | Herbicide | a_5,4_, a_5,5_, a_6,5_, a_7,5_, a_6,6_, a_7,6_, a_8,6_, a_6,7_, a_7,7_, a_8,7_, a_8,8_ | See matrix source | 0.793 | | 0.551 | | 83.01 | | 9.10 | | | 0.268 |
| *Alliaria petiolata* | Hardwood forests, Ohio, USA (λ_0_ = 1.451) | | | | | | | | | | | | |
|  | Mowing | a_3,2_, a_1,3_, | 71 | 0.466 | | 0.639 | | 67.9 | | 5.06 | | | 0.142 |
|  |  | a_2,3_ | 98 |  |  |  |  |  |  |  |  |  |  |
|  | Plateau | a_2,1_, a_1,3_, a_2,3_ | 97.42 | 0.301 | | 0.958 | | 79.25 | | 10.18 | | | 0.136 |
|  |  | a_3,2_ | 100 |  |  |  |  |  |  |  |  |  |  |
|  | Roundup | a_2,1_, a_2,3_ | 72.65 | 0.88 | | 0.958 | | 39.36 | | 6.93 | | | 3.145 |
|  |  | a_3,2_ | 80 |  |  |  |  |  |  |  |  |  |  |
|  |  | a_1,3_ | 90.37 |  |  |  |  |  |  |  |  |  |  |
|  | Triclopyr | a_2,1_, a_2,3_ | 92 | 0.31 | | 0.798 | | 78.66 | | 11.09 | | | 0.096 |
| *Ardisia elliptica* | Everglades National Park, Florida, USA (λ_0_ = 1.144) | | | | | | | | | | | | |
|  | Hand pulling | a_2,2_, a_3,2_, a_3,3_, a_4,3_, a_3,4_, a_4,4_, a_5,4_, a_4,5_, a_5,5_, a_6,5_ | 98 | 0.993 | | 0.457 | | 13.11 | | 10.24;  9.11-11.38 | | | 0.05 |
|  | Cut stump + basal bark | a_3,5_, a_4,5_, a_5,5_, a_6,5_, a_4,6_, a_5,6_, a_6,6_, a_7,6_ | 98 | 1.003 | | 0.155 | | 12.27 | | 64.37;  57.54-71.19 | | | 2.7 × 10^-3^ |
| *Carduus nutans* | Kybeyan, NSW, Australia (λ_0_ = 1.204) | | | | | | | | | | | | |
|  | Grazing | σ_1_, σ_2_, σ_3_, σ_4_ | 20 | 0.985 | | 3.19 | | 18.15 | | 48.14;  42.79-53.49 | | | 3.2 × 10^-4^ |
|  | Spray-grazing | σ_1_, σ_2_, σ_3_, σ_4_ | 99 | 0.04 | | 0.001 | | 96.7 | | 733.78;  730.57-736.99 | | | 1.47 × 10^-3^ |
|  | Herbicide | σ_1_, σ_2_, σ_3_, σ_4_ | 96 | 0.453 | | 0.022 | | 92.07 | | 680.83 | | | 0.022 |
|  | Midland and Argyll, North Island, New Zealand (λ_0_ = 2.688) | | | | | | | | | | | | |
|  | Grazing | σ_1_, σ_2_, σ_3_, σ_4_ | 20 | 2.271 | | 0.765 | | 15.5 | | 682.51 | | | 6.04 × 10^-4^ |
|  | Spray-grazing | σ_1_, σ_2_, σ_3_, σ_4_ | 99 | 0.157 | | 0.765 | | 94.16 | | 722.33 | | | 2.84 × 10^-3^ |
|  | Herbicide | σ_1_, σ_2_, σ_3_, σ_4_ | 96 | 0.335 | | 0.765 | | 87.54 | | 50.27 | | | 0.045 |
| *Centaurea stoebe subsp. micranthos* | Forested areas, Michigan, USA (λ_0_ = 1.332) | | | | | | | | | | | | |
|  | Prescribed fire | a_2,1_, a_3,2_, a_5,2_, a_5,3_, a_3,4_, a_4,4_, a_5,4,_ a_3,5_, a_4,5_ | See matrix source | 0.686 | | 0.67 | | 48.52 | | 121.2;  40.4-202 | | | 4.86 × 10^-3^ |
|  | Planting | a_2.1_, a_3,2_, a_4,2_, a_5,2_, a_3,3_, a_4,3_, a_5,3_, a_3,4_, a_4,4_, a_5,4_, a_3,5_, a_4,5_, a_5,5_ | 75 | 0.996 | | 0.256 | | 25.21 | | 160.39 | | | 1.6 × 10^-3^ |
|  | Milestone | a_3,2_, a_4,2_, a_5,2_, a_3,3_, a_4,3_, a_5,3_ | 99 | 0.501 | | 0.731 | | 62.36 | | 22.37;  18.33-26.41 | | | 0.041 |
|  |  | a_3,4_, a_4,4_, a_5,4_, a_3,5_, a_4,5_, a_5,5_ | 83 |  |  |  |  |  |  |  |  |  |  |
|  | Transline | a_3,2_, a_4,2_, a_5,2_, a_3,3_, a_4,3_, a_5,3_ | 99 | 0.501 | | 0.731 | | 62.36 | | 34.86;  30.82-38.89 | | | 0.026 |
|  |  | a_3,4_, a_4,4_, a_5,4_, a_3,5_, a_4,5_, a_5,5_ | 83 |  |  |  |  |  |  |  |  |  |  |
|  | Garlon 3A | a_3,2_, a_4,2_, a_5,2_, a_3,3_, a_4,3_, a_5,3_ | 90 | 0.52 | | 0.731 | | 61 | | 20.2;  16.16-24.24 | | | 0.042 |
|  |  | a_3,4_, a_4,4_, a_5,4_, a_3,5_, a_4,5_, a_5,5_ | 83 |  |  |  |  |  |  |  |  |  |  |
| *Cirsium vulgare* | Roadsides and pastures of eastern Nebraska, USA (λ_0_ = 1.54) | | | | | | | | | | | | |
|  | Hand pulling | a_3,2_, a_4,2_, a_3,3_, a_4,3_, a_4,4_ | 90 | 0.445 | | 0.582 | | 71.09 | | 30.35 | | | 0.027 |
|  | Roundup | a_3,2_, a_4,2_, a_3,3_, a_4,3_, a_4,4_ | 90 | 0.445 | | 0.582 | | 71.09 | | 14.95;  13.76-16.19 | | | 0.054 |
|  | Milestone | a_3,2_, a_4,2_, a_3,3_, a_4,3_, a_4,4_ | 94 | 0.209 | | 0.582 | | 86.44 | | 10.93;  9.71-12.14 | | | 0.08 |
|  | 2,4-D Ester | a_3,2_, a_4,2_, a_3,3_, a_4,3_, a_4,4_ | 78 | 0.666 | | 0.582 | | 56.75 | | 6.68;  5.46-7.89 | | | 0.105 |
|  | Tordon 22K | a_3,2_, a_4,2_, a_3,3_, a_4,3_, a_4,4_ | 91 | 0.498 | | 0.582 | | 67.67 | | 8.12;  8.95-11.36 | | | 0.097 |
|  | Weedmaster | a_3,2_, a_4,2_, a_3,3_, a_4,3_, a_4,4_ | 97 | 0.251 | | 0.582 | | 83.72 | | 5.76;  4.55-6.98 | | | 0.151 |
| *Cytisus scoparius* | Shoalhaven River in the Southern Tablelands, NSW, Australia (λ_0_ = 2.204) | | | | | | | | | | | | |
|  | Mulching | a_2,4_, a_3,4_, a_4,4_, a_5,4_, a_6,4_, a_7,4_, a_4,5_, a_5,5_, a_6,5_, a_7,5_, a_4,6_, a_5,6_, a_6,6_, a_7,6_, a_7,7_ | 85 | 1.579 | | 0.304 | | 28.35 | | 3502.77 | | | 1.6 × 10^-4^ |
| *Cytisus scoparius* | Cut stump | a_2,3_, a_3,3_, a_4,3_, a_5,3_, a_6,3_, a_7,3_, a_2,4_, a_3,4_, a_4,4_, a_5,4_, a_6,4_, a_7,4_, a_4,5_, a_5,5_, a_6,5_, a_7,5_, a_4,6_, a_5,6_, a_6,6_, a_7,6_, a_7,7_ | 99 | 1.451 | | 0.304 | | 34.13 | | 1167.59 | | | 5.7 × 10^-4^ |
|  | Spot spraying | a_2,1_, a_2,2_, a_3,2_, a_4,2_, a_5,2_, a_6,2_, a_2,3_, a_3,3_, a_4,3_, a_5,3_, a_6,3_, a_7,3_, a_2,4_, a_3,4_, a_4,4_, a_5,4_, a_6,4_, a_7,4_, a_4,5_, a_5,5_, a_6,5_, a_7,5_, a_4,6_, a_5,6_, a_6,6_, a_7,6_, a_7,7_ | 97 | 1.451 | | 0.304 | | 33.26 | | 2218.42 | | | 3.2 × 10^-4^ |
|  | Populations at the invasion edge in Joint Base Lewis-McChord, Washington, USA (λ_0_ = 2.016) | | | | | | | | | | | | |
|  | Prescribed fire | a_1,1_,a_2,1_,  a_2,2_, a_3,2_, a_4,2_, a_3,3_, a_4,3_, a_5,3_, a_6,3_, a_7,3_, a_4,4_, a_5,4_, a_6,4_, a_7,4_, a_4,5_, a_5,5_, a_6,5_, a_7,5_, a_6,6_, a_7,6_, a_7,7_ | 20  90 | 0.577 | | 0.838 | | 71.39 | | 7.31 | | | 0.168 |
|  | Mowing | a_1,3_, a_3,3_, a_4,3_, a_5,3_, a_6,3_, a_7,3_, a_1,4_, a_4,4_, a_5,4_, a_6,4_, a_7,4_, a_1,5_, a_4,5_, a_5,5_, a_6,5_, a_7,5_, a_1,6_, a_6,6_, a_7,6_, a_1,7_, a_7,7_ | 97.5 | 0.52 | | 0.603 | | 74.21 | | 123.83 | | | 9.57 × 10^-3^ |
|  | Spot Spraying | a_3,3_, a_4,3_, a_5,3_, a_6,3_, a_7,3_, a_4,4_, a_5,4_, a_6,4_, a_7,4_, a_4,5_, a_5,5_, a_6,5_, a_7,5_, a_6,6_, a_7,6_, a_7,7_ | 93 | 1.034 | | 0.441 | | 48.72 | | 20.64 | | | 0.04 |
|  | Populations at the invasion edge in Seattle City Parks, Washington, USA (λ_0_ = 1.24) | | | | | | | | | | | | |
|  | Mowing | a_1,3_, a_4,3_, a_5,3_, a_1,4_, a_5,4_, a_6,4_, a_1,5_, a_6,5_, a_7,5_, a_1,6_, a_7,6_, a_1,7_ | 90 | 0.739 | | 0.692 | | 40.41 | | 206.39 | | | 2.42 × 10^-3^ |
|  |  | a_3,3_, a_3,4_, a_4,4_, a_5,5_, a_6,6_, a_5,7_, a_6,7_, a_7,7_ | 25 |  |  |  |  |  |  |  |  |  |  |
|  | Hand clearing | a_3,3_, a_4,3_, a_5,3_, a_3,4_, a_4,4_, a_5,4_, a_5,5_, a_6,5_, a_7,5_, a_6,6_, a_7,6_, a_5,7_, a_6,7_, a_7,7_ | 80 | 0.771 | | 0.603 | | 37.82 | | 171.72 | | | 3.49 × 10^-3^ |
|  | Planting | a_2,1_, a_2,2_, a_3,2_, a_4,2_, a_3,3_, a_4,3_, a_5,3_, a_3,4_, a_4,4_, a_5,4_ | 90 | 0.889 | | 0.425 | | 28.29 | | 1324.2 | | | 3.6 × 10^-4^ |
| *Dipsacus sylvestris* | Kalamazoo County, Michigan USA (λ_0_ = 2.247) | | | | | | | | | | | | |
|  | Garlon 3A | a_3,1_, a_4,1_, a_5,1_, a_3,2_, a_3,3_, a_4,3_, a_5,3_, a_4,4_, a_5,4_, a_6,4_, a_5,5_, a_6,5_, a_3,6_, a_4,6_, a_5,6_ | 75 | 0.621 | | 0.964 | | 72.35 | | 23.72 | | | 0.069 |
|  | 2,4-D amine | a_3,1_, a_4,1_, a_5,1_, a_3,2_, a_3,3_, a_4,3_, a_5,3_, a_4,4_, a_5,4_, a_6,4_, a_5,5_, a_6,5_, a_3,6_, a_4,6_, a_5,6_ | 75 | 0.682 | | 0.964 | | 72.35 | | 24.72 | | | 0.066 |
| *Lespedeza cuneata* | Tyson Research Centre, Missouri, USA (λ_0_ = 22.454) | | | | | | | | | | | | |
|  | Chaparral | a_4,3_, a_4,4_, a_5,4_, a_4,5_, a_5,5_, a_6,5_, a_5,6_, a_6,6_ | 72.5 | 21.38 | | 0.065 | | 4.8 | | 7.62 | | | 0.14 |
|  | Cimarron | a_4,3_, a_4,4_, a_5,4_, a_4,5_, a_5,5_, a_6,5_, a_5,6_, a_6,6_ | 72.5 | 21.38 | | 0.065 | | 4.8 | | 4.71 | | | 0.226 |
|  | Crossbow | a_4,3_, a_4,4_, a_5,4_, a_4,5_, a_5,5_, a_6,5_, a_5,6_, a_6,6_ | 71 | 21.4 | | 0.065 | | 4.7 | | 13.97 | | | 0.075 |
|  | PastureGard | a_4,3_, a_4,4_, a_5,4_, a_4,5_, a_5,5_, a_6,5_, a_5,6_, a_6,6_ | 79.5 | 21.27 | | 0.065 | | 5.27 | | 9.61 | | | 0.121 |
|  | Remedy Ultra | a_4,3_, a_4,4_, a_5,4_, a_4,5_, a_5,5_, a_6,5_, a_5,6_, a_6,6_ | 71 | 21.4 | | 0.065 | | 4.7 | | 10.77 | | | 0.097 |
|  | Surmount | a_4,3_, a_4,4_, a_5,4_, a_4,5_, a_5,5_, a_6,5_, a_5,6_, a_6,6_ | 60 | 21.57 | | 0.065 | | 3.96 | | 6.58 | | | 0.134 |
| *Parkinsonia aculeata* | Populations spanning across the Northern Territory and Western Australia, Australia (λ_0_ = 1.253) | | | | | | | | | | | | |
|  | Double-chain pulling | a_4,5_, a_5,5_, a_6,5_, a_5,6_, a_6,6_, a_7,6_, a_6,7_, a_7,7_, a_8,7_, a_7,8_, a_8,8_, a_9,8_, a_8,9_, a_9,9_ | 24 | 1.161 | | 0.322 | | 7.36 | | 28.03 | | | 3.46 × 10^-3^ |
|  | Stick raking | a_4,5_, a_5,5_, a_6,5_, a_5,6_, a_6,6_, a_7,6_, a_6,7_, a_7,7_, a_8,7_, a_7,8_, a_8,8_, a_9,8_, a_8,9_, a_9,9_ | 40 | 1.105 | | 0.322 | | 11.82 | | 140.15 | | | 1.15 × 10^-3^ |
|  | Ellrott plough | a_4,5_, a_5,5_, a_6,5_, a_5,6_, a_6,6_, a_7,6_, a_6,7_, a_7,7_, a_8,7_, a_7,8_, a_8,8_, a_9,8_, a_8,9_, a_9,9_ | 90 | 0.968 | | 0.322 | | 22.78 | | 176.58 | | | 2.06 × 10^-3^ |
|  | Blade plough | a_4,5_, a_5,5_, a_6,5_, a_5,6_, a_6,6_, a_7,6_, a_6,7_, a_7,7_, a_8,7_, a_7,8_, a_8,8_, a_9,8_, a_8,9_, a_9,9_ | 90 | 0.968 | | 0.322 | | 22.78 | | 218.63 | | | 1.66 × 10^-3^ |
|  | Grubbing  via tractor | a_4,5_, a_5,5_, a_6,5_, a_5,6_, a_6,6_, a_7,6_, a_6,7_, a_7,7_, a_8,7_, a_7,8_, a_8,8_, a_9,8_, a_8,9_, a_9,9_ | 43 | 1.095 | | 0.322 | | 12.61 | | 165.37 | | | 1.05 × 10^-3^ |
|  | Soil-applied  Velpar | a_3,4_, a_4,4_, a_5,4_, a_4,5_, a_5,5_, a_6,5_, a_5,6_, a_6,6_, a_7,6_, a_6,7_, a_7,7_, a_8,7_, a_7,8_, a_8,8_, a_9,8_, a_8,9_, a_9,9_ | 97 | 0.705 | | 0.479 | | 43.76 | | 378.39 | | | 1.54 × 10^-3^ |
|  | Soil-applied  Grazon DS | a_3,4_, a_4,4_, a_5,4_, a_4,5_, a_5,5_, a_6,5_, a_5,6_, a_6,6_, a_7,6_, a_6,7_, a_7,7_, a_8,7_, a_7,8_, a_8,8_, a_9,8_, a_8,9_, a_9,9_ | 81 | 0.784 | | 0.479 | | 37.44 | | 266.28 | | | 1.83 × 10^-3^ |
|  | Basal bark application | a_3,3_, a_4,3_, a_3,4_, a_4,4_, a_5,4_, a_4,5_, a_5,5_, a_6,5_, a_5,6_, a_6,6_, a_7,6_, a_6,7_, a_7,7_, a_8,7_, a_7,8_, a_8,8_, a_9,8_, a_8,9_, a_9,9_ | 95 | 0.456 | | 0.637 | | 63.64 | | 561.65;  403.32-719.98 | | | 1.35 × 10^-3^ |
|  | Aerial foliar | a_3,2_, a_3,3_, a_4,3_, a_3,4_, a_4,4_, a_5,4_, a_4,5_, a_5,5_, a_6,5_ | 66 | 0.828 | | 0.616 | | 33.91 | | 215.82 | | | 2.36 × 10^-3^ |
|  | Flame thrower | a_3,3_, a_4,3_, a_3,4_, a_4,4_, a_5,4_, a_4,5_, a_5,5_, a_6,5_ | 83 | 0.822 | | 0.457 | | 34.43 | | 1353.98 | | | 3.5 × 10^-4^ |
| *Persicaria perfoliata* | Populations State Parks and Reserves in Pennsylvania and Delaware (λ_0_ = 0.67) | | | | | | | | | | | | |
|  | Pendulum aquacap *[Mar to Apr]* | **B_1_** – a_1,1_, a_2,1_, a_3,1_, a_4,1_ | 85 | 0.347 | | 0.509 | | 48.25 | | 39.5 | | | 7.36 × 10^-3^ |
|  | Plateau & Oust  *[Mar to Apr]* | **B_1_** – a_1,1_, a_2,1_, a_3,1_, a_4,1_ | 85 | 0.347 | | 0.509 | | 48.25 | | 26.37 | | | 0.011 |
|  | Hand pulling  *[May-mid Jun]* | **B_2_** - a_1,1_, a_1,2_, a_1,3_ | 87.5 | 0.364 | | 0.509 | | 45.63 | | 18.89 | | | 0.015 |
|  | Garlon 3A  *[May – Jun]* | **B_2_** - a_1,1_, a_1,2_, a_1,3_, a_2,4_ | 85 | 0.347 | | 0.509 | | 48.16 | | 26.94 | | | 0.011 |
|  | Hand pulling  *[Jun - Jul]* | **B_3_** - a_1,1_ | 87.5 | 0.337 | | 0.509 | | 49.72 | | 18.89 | | | 0.016 |
|  | Garlon 3A  *[Jun - Jul]* | **B_3_** - a_1,1_ | 85 | 0.347 | | 0.509 | | 48.13 | | 26.94 | | | 0.011 |
| *Pinus nigra*  subsp. *laricio* | Mt Barker, South Island, New Zealand (λ_0_ = 5.243) | | | | | | | | | | | | |
|  | Grazing | a_2,1_ | 90 | 3.075 | | 0.244 | | 41.36 | | 331.72 | | 3.66 × 10^-3^ | |
|  | Fertiliser | a_2,1_ | 50 | 4.437 | | 0.244 | | 15.37 | | 90.39 | | 3.48 × 10^-3^ | |
|  | Hand pulling | a_2,1_ | 98 | 2.205 | | 0.244 | | 57.94 | | 1.81 | | 7.09 × 10^-3^ | |
|  | Hand tools | a_2,2_, a_3,2_ | 98 | 2.023 | | 0.285 | | 61.42 | | 49.72 | | 0.694 | |
|  | Ground foliar | a_2,1_, a_2,2_, a_3,2_, a_4,3_ | 70 | 2.294 | | 0.713 | | 56.24 | | 90.39 | | 0.029 | |
|  | Aerial foliar | a_2,1_, a_2,2_, a_3,2_, a_4,3_ | 70 | 2.294 | | 0.713 | | 56.24 | | 451.97 | | 5.79 × 10^-3^ | |
|  | Scrub-bar | a_2,2_, a_3,2_ | 98 | 2.023 | | 0.285 | | 61.42 | | 90.39 | | 0.016 | |
|  | Basal bark application | a_2,2_, a_3,2_, a_4,3_ | 90 | 1.951 | | 0.285 | | 27.62 | | 24.88 | | 0.089 | |
|  | Soil uptake | a_2,2_, a_3,2_ | 70 | 3.795 | | 0.469 | | 62.79 | | 131.07 | | 7.98 × 10^-3^ | |
|  | Ring barking | a_4,3_, a_4,4_ | 20 | 4.994 | | 0.226 | | 4.75 | | 90.39 | | 2.62 × 10^-3^ | |
|  | Chainsaw | a_4,3_, a_4,4_ | 98 | 3.473 | | 0.226 | | 33. 76 | | 20.34 | | 5.71 × 10^-3^ | |
|  | Cut stump | a_4,3_, a_4,4_ | 90 | 3.73 | | 0.226 | | 28.86 | | 135.59 | | 7.87 × 10^-3^ | |
|  | Stem poisoning | a_4,3_, a_4,4_ | 90 | 3.73 | | 0.226 | | 28.86 | | 361.58 | | 2.95 × 10^-3^ | |
| *Prunus serotina* | Light conditions, Compiegne Forest, France (λ_0_ = 1.162) | | | | | | | | | | | | |
|  | Felling | a_6,5_, a_7,6_, a_8,7_, a_9,8_, a_10,9_, a_11,10_, a_11,11_ | 99.9 | 0.018 | | 0.761 | | 98.49 | | 14,036.6;  10,917.3-17,155.8 | | 6.29 × 10^-5^ | |
|  | Cutting with some debarking | a_7,6_, a_8,7_, a_9,8_, a_10,9_, a_11,10_, a_11,11_ | 20 | 0.999 | | 0.697 | | 13.93 | | 701.83;  155.96-1,247.7 | | 2.3 × 10^-4^ | |
|  | Ring barking | a_7,6_, a_8,7_, a_9,8_, a_10,9_, a_11,10_, a_11,11_ | 74.95 | 0.999 | | 0.697 | | 13.93 | | 265.14;  62.38-467.89 | | 6.1 × 10^-3^ | |
|  | Glyphosate | a_5,4_, a_6,5_, a_7,6_, a_8,7_ | 99.9 | 0.506 | | 0.697 | | 56.44 | | 5,848.57;  3899.05-7,798.1 | | 1 × 10^-5^ | |
|  | Triclopyr | a_5,4_, a_6,5_, a_7,6_, a_8,7_ | 90 | 0.99 | | 0.259 | | 14.76 | | 5,848.57;  3899.05-7,798.1 | | 5.13 × 10^-5^ | |
|  | Cut stump | a_7,6_, a_8,7_, a_9,8_, a_10,9_, a_11,10_, a_11,11_ | 90 | 0.99 | | 0.259 | | 14.76 | | 2,074.29;  249.54-3,899.05 | | 1.3 × 10^-5^ | |
|  | Hand pulling | a_4,3_, a_5,4_, a_6,5_, a_7,6_ | 99.9 | 0.311 | | 0.697 | | 73.23 | | 185.14;  123.43-246.86 | | 1.2 × 10^-4^ | |
| *Rubus armeniacus* | Low-density populations in forested areas, OR (λ_0_ = 1.148) | | | | | | | | | | | | |
|  | Hand clearing | a_1,1_, a_2,1_, a_3,2_, a_4,3_, a_3,4_, a_4,4_ | 99 | 0.022 | | 0.975 | | 98.1 | | 1019.81 | | 1.09 × 10^-3^ | |
|  | Spot spray | a_1,1_, a_2,1_, a_3,2_, a_1,3_, a_4,3_, a_3,4_, a_4,4_ | 95 | 0.057 | | 1 | | 95 | | 185.14;  123.43-246.86 | | 5.89 × 10^-3^ | |
|  | Weed-eater | a_2,1_, a_3,2_, a_1,3_, a_3,4_, a_4,4_ | 90 | 0.348 | | 0.548 | | 69.71 | | 88.87 | | 6.38 × 10^-3^ | |

^1^Grasslands National Park; ^2^Everglades and Dry Torugas National Parks, Florida; ^3^Cooma-Monaro Shire Council, New South Wales; ^4^NSW Department of Primary Industries; ^5^Massey University, New Zealand; ^6^Horizons Regional Council, New Zealand; ^7^Michigan Department of Natural Resources; ^8^Nebraska Department of Agriculture; ^9^University of Nebraska – Lincoln; ^10^Southern Rivers Catchment Management Authority, New South Wales; ^11^Joint Base Lewis-McChord, Washington; ^12^Centre for Natural Lands Management, Washington; ^13^Seattle Parks and Recreation; ^14^University of Missouri; ^15^Missouri Department of Conservation; ^16^Department of Management and Sustainable Use, Belgium; ^17^Research Institute for Nature and Forest, Belgium; ^18^CSIRO Ecosystem Sciences, Queensland; ^19^Pilbara Mesquite Management Committee, Western Australia; ^20^Department of Environment and Conservation, Western Australia; ^21^New Zealand Wilding Conifer Management Group; ^22^Department of Conservation, New Zealand; ^23^U.S. Forest Service; ^24^Oregon Department of Agriculture.
