# Supplementary material for: Prioritizing management actions for invasive populations using cost, efficacy, demography and expert opinion for 14 plant species world‐wide
Source: J Appl Ecol. 2016 Feb 22;53(2):305–16. doi: 10.1111/1365-2664.12592 (PMC4949517; doi:10.1111/1365-2664.12592)
Supplement: Supplementary file 5 — Appendix S5. Table of symbols and definitions of model parameters and management objectives. [file JPE-53-305-s005.docx]

**Appendix S5.** Table of symbols and definitions of model parameters and management objectives.

| Name | Symbol | Definition |
| --- | --- | --- |
| Management strategy | *x* | Management actions used to control the invasive plant species at the site where the population study was conducted |
| Matrix element | *a_ij_* | Matrix element of transition from life stage *j* to *i* |
| Population growth rate | λ | Asymptotic population growth rate of unmanaged (λ_0_) or managed (λ_x_) populations |
| Sensitivity* | *s_ij_* | The additive response of λ to changes in transition, a*_ij_* |
| Elasticity* | *e_ij_* | The proportional response of λ to proportional changes in transition, *a_ij_* |
| Matrix element efficacy | *f_ij,x_* | Studied or observed efficacy of management action *x* at reducing the vital rates of individuals within matrix transition *ij* |
| Cost | *c_x_* | Cost (US$) of implementing management action *x* per hectare |
| Efficacy [of management] | Δλ*_x_* | Total reduction in population growth rate with management action *x* |
| Marginal cost^+^ | *m_ij,x_* | Cost of management action (*c_x_*) in respect to its efficiency (*f_ij_)* at altering the vital rates of matrix element *a_ij_* |
| Cost-effectiveness  (Economic sensitivity analysis)^+^ | *g_x_* | The cost of a small change to population growth |

(*Caswell 2001, ^+^Baxter et al. 2006)
