# Supplementary material for: Prioritizing management actions for invasive populations using cost, efficacy, demography and expert opinion for 14 plant species world‐wide
Source: J Appl Ecol. 2016 Feb 22;53(2):305–16. doi: 10.1111/1365-2664.12592 (PMC4949517; doi:10.1111/1365-2664.12592)
Supplement: Supplementary file 6 — Appendix S6. Responses of survey analysis. [file JPE-53-305-s006.docx]

**Appendix S6. Responses of survey analysis** and number of manager ranks provided (e.g. some managers provided qualitative feedback only, without ranks). Managers were provided with a list of all actions that had available management data and that were used in our analysis; they were asked to rank these actions, where possible, and provide a brief justification for why actions received those ranks. Content analysis identified categories of words that managers used to justify their ranks (cost, efficacy, demographic considerations, environmental impacts and time consumption). Other comments not captured in the content categories are also given. Capital letters denote individual manager identities; if a column or comment receives that letter then the manager considered that factor when ranking actions.

| **Species** | **Responses/ranking** | **Alignment with metrics** | **Cost** | **Efficacy** | **Demographic considerations** | **Environmental impacts** | **Time consumption (long and short term)** | **Other comments** |
| --- | --- | --- | --- | --- | --- | --- | --- | --- |
| *Agropyron cristatum* | 1/1 | Efficacy (A) |  | A | A |  |  | Dependency on weather and timing (A) |
| *Alliaria petiolata* | 1/0 |  | B | B | B | B |  |  |
| *Carduus nutans* | 6/6 | Cost effectiveness (C,D,E,F,G,H) | F | D,G,H | C,D,G | G | H | Viability that livestock will eat thistles (F,H)  Herbicide resistance (C)  Targets multiple species (E,F) |
| *Cirsium vulgare* | 2/2 |  | I | I | I |  |  | Social impacts of herbicide (I)  Viability with density (I, J)  Broad applicability to different sites (J) |
| *Cytisus scoparius*  (NSW) | 1/1 |  |  | K | K | K | K | Viability with density (K) |
| *Cytisus scoparius* (Prairie fields) | 1/1 |  |  | L | L | L |  | Viability with density (L) |
| *Cytisus scoparius*  (City Parks) | 2/2 | Cost and cost effectiveness  (only for M) | M | M,N | M,N | N | M,N | Viability with density (M) |
| *Lespedeza cuneata* | 1/1 |  | O | O,P | O |  |  | Residual effects on future seedlings (O)  Viability on the short-term (N) |
| *Parkinsonia*  *aculeata* | 1/1 |  | Q |  | Q | Q | Q | Risk of failure/Difficult of use (Q)  Legal status (Q)  Need for skilled operators (Q)  Off-site costs (Q)  Viability as a follow-up method (Q) |
| *Persicaria perfoliata* | 3/2 |  |  | S,U |  | S,T,U | T | Recruitment of volunteers (S)  Park visitor appeal towards method (S)  Viability as a follow-up method (U) |
| *Pinus nigra* (subsp. *Laricio*) | 1/1 |  | V | V | V |  | V | Topography at study site (V) |
| *Prunus*  *serotina* | 1/1 |  | W | W |  | W |  | Difficulty of method (W) |
| *Rubus armeniacus* | 3/3 | Elasticity  (X,Y,Z) | X,Z | X,Y,Z | Y | Z | X,Z | Difficulty of method (X)  Long-term effects on population(Y) |
