# Supplementary material for: Prioritizing management actions for invasive populations using cost, efficacy, demography and expert opinion for 14 plant species world‐wide
Source: J Appl Ecol. 2016 Feb 22;53(2):305–16. doi: 10.1111/1365-2664.12592 (PMC4949517; doi:10.1111/1365-2664.12592)
Supplement: Supplementary file 7 — Appendix S7. Agropyron cristatum. [file JPE-53-305-s007.docx]

**Appendix S7.** ***Agropyron cristatum***

Fact sheet on the management of *Agropyron cristatum* populations in Grasslands National Park, Canada.

Methods

We used matrix models developed for populations of *Agropyron cristatum* under three conditions – herbicide-treated (glyphosate), clipped, and no treatment - in Grasslands National Park, Canada (Hansen & Wilson 2006; Hansen 2007). For the construction of the matrix model, the life cycle of *Agropyron cristatum* was partitioned into six stage-classes: seed bank, juvenile (1 year old; one tiller) and four tussock classes based on size (1-46, 47-77, 78-140, and >140 tillers).

We obtained management data for three control actions – grazing, mowing, and herbicide - from managers at Grasslands National Park. We used their five population matrices for our study with the assumption that management efficacy for grazing and mowing practices could be extracted from the clipped population matrix, despite probable but unquantifiable differences in effects between these management techniques. We included the direct effects of mowing and grazing - reduction in fertility and growth – and the direct effects of herbicide spray – reduction in survival and growth. Using the managed matrices, we calculated marginal cost of management action *x* by dividing the total cost per hectare (*c_x_*) by the proportional reduction in each transition value affected:

$$m_{ij,x}= \frac{c_{x}}{a_{ij}- b_{ij}}$$

where *a_ij_* is the matrix element from an unmanaged population matrix and *b_ij_* is the matrix element from the published managed population matrix. We obtained the most recent management cost estimates from managers at Grasslands National Park; however, sheep grazing is cost-neutral to Grasslands National Park due to being mutually beneficial for both the farmers and the park. We set the cost of grazing to US$0.01, so that metrics for this management action could be calculated for our analyses. Native seed addition (seven grasses and wildflowers) is another action used in Grasslands National Park to assist with the long-term control of invasive grass species via competition; however, we were unable to properly estimate the effects of native seed addition on the demography of *Agropyron cristatum*, so this management action was omitted. We converted all cost estimates to same units of measurement (cost per hectare) and to US dollars (17 February 2012, www.oanda.com) to compare data across species and sites. See Methods section main text for more details on data analysis.

Results

When looking among years, management actions for *Agropyron cristatum* received the same preference order for all management proxies (elasticity, cost and efficacy) and objectives (any reduction in λ, and λ<1). However, all actions became more cost-effective the greater the time interval between management events. For example, these management actions were theoretically more cost-effective after three years without management (2004-2005) than one year without management (2002-2003).

All management actions could theoretically achieve local extinction by reducing population growth rate below 1 across 2002-2005. However, this assumes that the matrix model for clipped populations could be extrapolated for both grazing and mowing. Sheep grazing was the most cost-effective due to it being cost neutral, while herbicide the least cost-effective.

Several managers said that many of these actions are not viable individually, but rather that an ongoing integrated management would be required to observe significant long-term effects on *Agropyron cristatum* populations within the park. One manager was able to rank these management actions based on their individual merits, despite their inefficiency independently. Herbicide was their top-ranked method; this was justified based on its demographic targets and efficacy. Using grazing as a means of controlling *Agropyron cristatum* was said to be temporally variable, depending on favorable conditions for both the park and farmers, and was seen as more of an investment on the long-term. The manager rankings aligned with efficacy ranks, yet efficacy provided both mowing and grazing with the same rank unlike the manager ranks. This highlights the need for coarser efficacy estimates in order to properly assess the differences between actions.

References

Hansen, M. and S. Wilson. (2006). Is management of an invasive grass *Agropyron cristatum* contingent on environmental variation? *Journal of Applied Ecology*, **43**, 269-280.

Hansen, M. J. (2007). Evaluating management strategies and recovery of an invasive grass (*Agropyron cristatum*) using matrix population models. *Biological Conservation*, **140**, 91-99.
