# Supplementary material for: Prioritizing management actions for invasive populations using cost, efficacy, demography and expert opinion for 14 plant species world‐wide
Source: J Appl Ecol. 2016 Feb 22;53(2):305–16. doi: 10.1111/1365-2664.12592 (PMC4949517; doi:10.1111/1365-2664.12592)
Supplement: Supplementary file 8 — Appendix S8. Alliaria petiolata. [file JPE-53-305-s008.docx]

**Appendix S8. *Alliaria petiolata***

Fact sheet for management of *Alliaria petiolata* populations in hardwood forests of Ohio, USA.

Methods

Davis *et al.* (2006) constructed a matrix population model for three population densities of biennial invasive herb, *Alliaria petiolata* or commonly known as Garlic Mustard*,* in second growth hardwood forests located on Ridges Land Laboratory in Ohio from existing empirical, demography studies. These matrix model partitions the life history of *Alliaria petiolata* into three life stages based on age and developmental characteristics: seed bank, rosettes (first year), and reproductive adults (second year). For our analyses, we only used the low-density matrix because of the density-dependence assumption of population projection matrix models.

We contacted managers from nature reserves and government departments, but no managers were able to provide the appropriate management data for *Alliaria petiolata* for our analyses. We used a combination of search terms – *Alliaria petiolata*, Garlic Mustard, cost, efficacy, and control – for Web of Science, Google Scholar and Google to find appropriate management data. We found generic cost data for some common actions used to control broadleaf plants from reports within the grey literature (Tu, Hurd & Randall 2001; Loux 2008; Loux *et al.* 2013) as well as reported efficacy values specific to *Alliaria petiolata* control (Nuzzo 1991; Pandian & Renz 2008). Since the same action can have varying effects on different species, the available management efficacy specific to *Alliaria petiolata* limited our analyses to only four management actions - Mowing, Roundup, Plateau, and Triclopyr. Despite there being no biocontrol agents currently used to control populations, there is active research into promising agents for *Alliaria petiolata.* All cost estimates were converted to present value for 2012 (BLS 2015). See Methods section of main text for more details on data analysis.

Results

Efficacy and elasticity analyses produced different management ranks signifying the importance of accounting for the effects of management on transition rates; a caveat to the elasticity analysis (de Kroon, van Groenendael & Ehrlen 2000). Despite Plateau and Roundup having the same demographic targets, efficacy analysis found that Plateau was most effective action at controlling *Alliaria petiolata* than Roundup, which was the least effective out of all actions. Despite not being the cheapest, Roundup became the most cost-effective action by at least 20 fold. Out of the management proxies, cost aligned the most with cost-effectiveness for this invasive herb supporting that management cost contributes the most toward cost-effectiveness, particularly for short-lived plants.

Since all management actions can theoretically achieve local extinction, accounting for the absolute reduction in population growth rate resulting from management would be unnecessary. Therefore, the cost could be used as a proxy for identifying suitable actions for controlling this population of *Alliaria* *petiolate* under financial constraints or for both management objectives (any reduction in λ, or λ<1).

Manual removal by volunteers is a common approach for controlling *Alliaria petiolata* in many nature reserves of Ohio; however, the lack of management data means we were unable to include this action within our analyses. From manager responses to our survey, none were able to provide ranks for these methods and one manager provided reasons for selecting different actions to control *Alliaria petiolata* in forested areas*.* For this manager, the main decision-making factors were cost, effectiveness, effects on demography, and environmental impacts of methods since this invasive herb is commonly found in aquatic areas.

References

Bureau of labor statistics. (2015) CPI Inflation calculator. Division of Consumer Prices and Price Indexes, Washington DC. Available from: <http://www.bls.gov/data/inflation_calculator.htm>

Davis, A.S., Landis, D.A., Nuzzo, V., Blossey, B., Gerber, E., & Hinz, H.L. (2006). Demographic models inform selection of biocontrol agents for garlic mustard (*Alliaria petiolata*). *Ecological Applications*, **16**, 2399-2410.

de Kroon, H., van Groenendael, J., & Ehrlen, J. (2000). Elasticities: A Review of Methods and Model Limitations. *Ecology*, **81**, 607-618.

Loux, M. (2008) Trying to Make Sense of the Soybean Seed and Herbicide Costs. *Crops*, <http://ocj.com/wp-content/uploads/2011/06/CCrops15-20.pdf>, version: December 2008

Loux, M.M., Doohan, D., Dobbels, A.F., & Reeb, B. (2013) Weed Control Guide for Ohio and Indiana. Ohio State University Extension. <http://agcrops.osu.edu/specialists/weeds/specialist-links/2010%20Weed%20Control%20Guide.pdf>, version: November 2013.

Nuzzo, V.A. (1991). Experimental control of garlic mustard [*Alliaria petiolata* (Bieb.) Cavara & Grande] in northern Illinois using fire, herbicide, and cutting. *Natural Areas Journal*, **11**, 158-167.

Pandian, V., & Renz, M.J. (2008). Evaluation of Garlic Mustard Control with Spring and Fall Herbicide Application. *North Central Weed Science Society Proc*, 63-73. Available from: <http://www.ncwss.org/proceed/2008/abstracts/073.pdf> and <http://buffalo.uwex.edu/files/2010/06/Evaluation-of-Garlic-Mustard-Control-with-Spring-and-Fall-He.pdf>

Tu, M., Hurd, C., & Randall, J.M. (2001). Weed Control Methods Handbook: Tools and Techniques for Use in Natural Areas. The Nature Conservancy. <http://www.invasive.org/gist/products/handbook/methods-handbook.pdf>, version: April 2001.
