# Supplementary material for: Prioritizing management actions for invasive populations using cost, efficacy, demography and expert opinion for 14 plant species world‐wide
Source: J Appl Ecol. 2016 Feb 22;53(2):305–16. doi: 10.1111/1365-2664.12592 (PMC4949517; doi:10.1111/1365-2664.12592)
Supplement: Supplementary file 9 — Appendix S9. Ardisia elliptica. [file JPE-53-305-s009.docx]

**Appendix S9.** ***Ardisia elliptica***

Fact sheet for management of *Ardisia elliptica* populations in Everglades National Park in Florida, USA.

Methods

Koop and Horvitz (2005) developed matrix models for *Ardisia elliptica* at five sites in Everglades National Park in Florida, USA from 1999 to 2001. These five sites represent two types of hardwood forest (hardwood forest and forest edge) and three types of disturbed forest (transition forest, Ardisia thicket, and Schinus thicket). For the construction of this stage-based model, the life history of *Ardisia elliptica* was partitioned into eight stages - seedling, small juvenile, medium juvenile, large juvenile, pre-reproductive, small adult and large adult - based on size and developmental characteristics (Koop & Horvitz 2005).

We contacted managers at Everglades National Park who informed us that *Ardisia elliptica* has not been a problem weed at the park since large-scale management events that occurred in the late 1980s and early 1990s. However, we were able to obtain management data for *Ardisia elliptica* assembled from once-off management of low-density infestation of small plants (less than 1 m in height, trunk at base <2.5 cm in diameter) on January 12, 2012 (H. Cooley, pers. comm., 27 January 2012) with only two distinct interventions: hand pulling and both cut stump and basal bark application, which were combined for our analyses because management data were unable to be distinguished. Due to this management situation, the cost and efficacy of cut stump and basal bark application were unable to be distinguished from this management event and, therefore, were treated as one method for our study. Additionally, our study assumed that these cost-estimates could be extrapolated to all five study sites. Since *Ardisia elliptica* is usually under control within the park without intervention, managers were unable to rank these actions. See Methods section of main text for more details on data analysis.

Results

We found that these management actions were most cost-effective at managing *Ardisia elliptica* in transition and hardwood forests in Everglades National Park, Florida, and least cost-effective in Schinus thickets. For most populations across the years, these two actions received mostly the same order across all management proxies (elasticity, efficacy, and cost) and objectives (any reduction in λ, or λ<1). Despite hand pulling being the cheapest action and cost ranges not overlapping (Fig 9.1), cut stump and basal bark was the most cost-effective action in Ardisia thickets in 2001. In all other years and sites, hand pulling was the most cost-effective action. These findings suggest that cost cannot always be used as a substitute for the cost-effectiveness analysis, and demography also needs to be considered when making suitable recommendations for management.


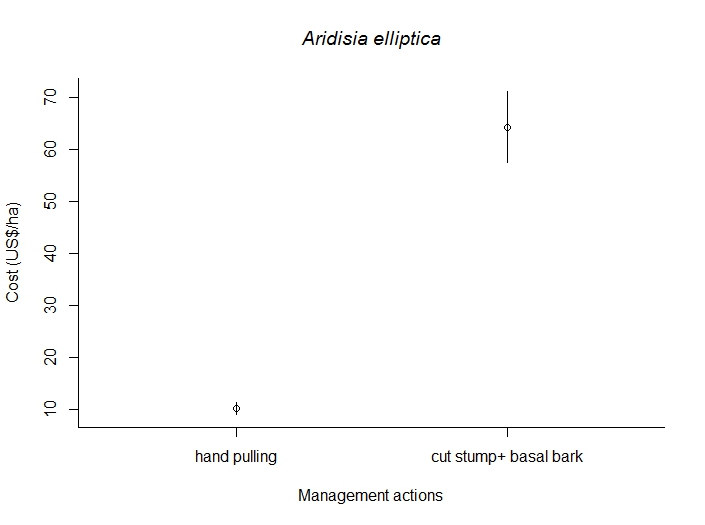


**Figure 9.1.** Cost ranges (US$ per ha) for the two management actions, hand pulling and cut stump + basal bark, used to control *Ardisia elliptica* in Everglades National Park, Florida, USA. Circles represent mean cost values, and lines represent the range in cost estimates for each action.

Managers were unable to rank these two methods used in our study. The reasoning being that both methods are usually implemented in an integrated way within the park meaning managers were unable to rank them independently, and *Ardisia elliptica* is not problematic weed meaning that management invention is rarely necessary within Everglades National Park.

References

Koop, A. & Horvitz, C. (2005). Projection matrix analysis of the demography of an invasive, nonnative shrub (Ardisia elliptica). *Ecology*, **86**, 2661-2672.
