# Supplementary material for: Prioritizing management actions for invasive populations using cost, efficacy, demography and expert opinion for 14 plant species world‐wide
Source: J Appl Ecol. 2016 Feb 22;53(2):305–16. doi: 10.1111/1365-2664.12592 (PMC4949517; doi:10.1111/1365-2664.12592)
Supplement: Supplementary file 10 — Appendix S10. Carduus nutans. [file JPE-53-305-s010.docx]

**Appendix S10.** ***Carduus nutans***

Fact sheet for management of *Carduus nutans* populations on the North Island of New Zealand and near Kybeyan in New South Wales, Australia.

Methods

Shea *et al.* (2010) developed matrix models parameterized for populations of *Carduus nutans* near Kybeyan in New South Wales and on the North Island of New Zealand. The matrix model for *Carduus nutans* had four life stages: seed bank, small rosettes (AU: <20.8cm^2^; NZ: <83.1 cm^2^), medium-sized rosettes (AU: 20.8-43.4 cm^2^; NZ: 83.1-175 cm^2^), and large rosettes (AU: >43.4 cm^2^; NZ: >175 cm^2^). Along with these matrix models, Shea *et al.* (2010) also published efficacy values for herbicide, spray-grazing and crash grazing summarized from the literature as well as the underlying vital rates of the matrix models. Because management actions can have effects on different individual underlying vital rates, we looked at the effect of management on the underlying survival parameters that were published compared to the whole transition rates. We found no difference between marginal cost values when altering the underlying survival parameter compared to the whole transition rate for *Carduus nutans*, so we used the transition rate to be consistent across all species.

We were able to obtain estimate cost of crash grazing in New South Wales assuming that farmers do not own a portable water tank (cost for 25,000L tank) and that no fencing was already established on the property (G. Meaker, pers. comm., 8 February 2012). Because of the unavailability of grazing data in New Zealand, we assumed that these costs could be extrapolated to crash grazing in New Zealand. Management data was obtained from five managers, respectively. Additionally, the cost-estimates of control methods used in New Zealand and Australia were converted to the US dollar (17 February 2012, www.oanda.com) to compare data across species and sites. Biological control agents are commonly and successfully used to control *Carduus nutans* populations in both Australia and New Zealand, but it was excluded from our study because of the differences in cost structure and time-scale of management effects. See Methods section of main text for more details on data analyses.

Results

All three management actions received the same elasticity value in both Australia and New Zealand, whereas efficacy analysis provided complete discrimination between management actions. None of the management proxies aligned with cost-effectiveness. Management actions received the same rank in both Australia and New Zealand according to cost-effectiveness, yet actions were more cost-effective at controlling populations in New Zealand. Not only was crash grazing was the least cost-effective for both populations, this action was also unable to achieve declining populations in New Zealand.

All six managers ranked actions in the same order as cost-effectiveness; herbicide ranked first and crash grazing ranked last. One manager ranked both spray-grazing and crash grazing the same due to herbicide resistance for spray-grazing and overgrazing opening up the paddock to seed germination for crash grazing, but this ranking did not conflict with the cost-effectiveness. According to the survey responses, the key considerations in both Australia and New Zealand were cost, demographic targets, effectiveness on the short- (residual effects on the next generation) and long-term (herbicide resistance), and their ability to target multiple invasive plant species.

References

Shea, K., Jongejans, E., Skarpaas, O., Kelly, D., & Sheppard, A.W. (2010). Optimal management strategies to control local population growth or population spread may not be the same. *Ecological Applications*, **20**, 1148-1161.
