# Supplementary material for: Prioritizing management actions for invasive populations using cost, efficacy, demography and expert opinion for 14 plant species world‐wide
Source: J Appl Ecol. 2016 Feb 22;53(2):305–16. doi: 10.1111/1365-2664.12592 (PMC4949517; doi:10.1111/1365-2664.12592)
Supplement: Supplementary file 11 — Appendix S11. Centaurea stoebe. [file JPE-53-305-s011.docx]

**Appendix S11.** ***Centaurea stoebe***

Fact sheet for management of *Centaurea stoebe* populations in forested areas of Michigan, USA.

Methods

We used a matrix developed for invasive herb *Centaurea stoebe* (formerly, *Centaurea maculata*), which was parameterized for populations in undeveloped prairie and oak savanna remnants near Augusta, Michigan. The life stage partitioning of *Centaurea stoebe* into five classes – seed bank, juvenile, non-reproductive individuals, small adults and large adults - was constructed from developmental characteristics (Emery & Gross 2005).

Emery and Gross (2005) also developed matrix models for populations subjected to seasonal annual and biennial fires, where they found that annual summer fires was the most effective at reducing population growth rates. We used the annual summer fire matrix to calculate the marginal cost similar to other management actions for *Agropyron cristatum* (S7). Using the annual summer fire matrix, we calculated marginal cost by dividing the total cost per hectare (*c_x_*) by the proportional reduction in each transition value affected:

$$m_{ij,x}= \frac{c_{x}}{a_{ij}- b_{ij}}$$

Where *a_ij_* is the matrix elements from the unmanaged population matrices and *b_ij_* from the managed population matrices. We obtained the remaining management data from two managers at the Michigan Department of Natural Resources. Management data were found for prescribed fire, planting of native seeds, and three herbicides: Milestone, Transline, and Garlon 3A. A number of successful biocontrol agents have been used to control populations of *Centaurea stoebe*; however, these actions were excluded due to the differences in cost structure and time-scale effects compared to local management actions. See Methods section of main text for more details on data analysis.

Results

Efficacy analysis ranked Milestone and Transline as the most effective actions, yet Transline dropped to third rank for cost-effectiveness. Cost-effectiveness ranks aligned with cost ranks for management actions for *Centaurea stoebe* in forested areas of Michigan. Since all management actions were able to achieve a declining population, management cost could be used as a substitute for cost-effectiveness analysis, if a demographic model cannot be constructed due to limited resources or the need for swift response to new infestations.

Unfortunately, we received no manager responses from our survey for *Centaurea stoebe* management in forested areas of Michigan, USA.

References

Emery, S. & Gross, K. (2005). Effects of timing of prescribed fire on the demography of an invasive plant, spotted knapweed Centaurea maculosa. *Journal of Applied Ecology*, **42**, 60-69.
