# Supplementary material for: Prioritizing management actions for invasive populations using cost, efficacy, demography and expert opinion for 14 plant species world‐wide
Source: J Appl Ecol. 2016 Feb 22;53(2):305–16. doi: 10.1111/1365-2664.12592 (PMC4949517; doi:10.1111/1365-2664.12592)
Supplement: Supplementary file 12 — Appendix S12. Cirsium vulgare. [file JPE-53-305-s012.docx]

**Appendix S12.** ***Cirsium vulgare***

Fact sheet for management of low-density populations of *Cirsium vulgare* along roadsides and in pastures of Nebraska, USA.

Methods

We used a matrix model developed for low-density populations of a perennial herb, *Cirsium vulgare* (commonly known as Bull Thistle), in pastures and along road-sides in eastern Nebraska ([Tenhumberg *et al.* 2008](#_ENREF_3)). This matrix partitioned its life history into four stages – seed bank, small, medium and large – based on developmental characteristics (Bullock, Hill & Silvertown 1994; Tenhumberg *et al.* 2008).

We used both data from managers and reported estimates from the grey literature. We used a combination of search terms – *Cirsium vulgare*, cost, efficacy, management, and control – for Web of Science, Google Scholar and Google to find appropriate management data. We accrued all herbicide cost data, apart from Roundup, from the Guide for Weed Management for Nebraska developed by University of Nebraska, Lincoln (UNL 2011, see Ditches and Roadsides section). We adjusted these costs to herbicide application rates from the reported efficiency values (Doll 2000, Dow AgroSciences 2011, Deneke *et al.* 2012). We obtained the additional costs of herbicide application, such as labour and equipment, from researchers at the Department of Agronomy and Horticulture at the University of Nebraska-Lincoln.

For *Cirsium vulgare* along roadsides and pastures in Nebraska, we found data for six management actions: hand pulling, Glyphosate, Milestone, 2,4-D Ester, Tordon 22K, and Weedmaster. Several biocontrol agents have been released in Nebraska and the neighbouring states to control many invasive *Cirsium spp.*; some agents have impacts on *Cirsium vulgare* populations. Due to the differences in cost structure and time-scale effects to local management actions, biocontrol actions were excluded from our study. See Methods section of main text for more details on data analysis.

Results

The management of *Cirsium vulgare* along roadsides and in pastures of Nebraska is a perfect illustration of elasticity analysis unable to discriminate between management actions, despite management actions having varying effects on vital rates as demonstrated in the efficacy analysis. All actions targeted the same life stage transitions and, consequently, received the same elasticity value. Short-lived species often have a simpler life history than long-lived plants meaning that it is easier for management actions to target multiple life stages. Therefore, demography may play less of a role in determining suitable management for short-lived species.

Despite efficacy analysis discriminating among actions, management cost was the only proxy that aligned almost completely with cost-effectiveness. Weedmaster, a type of herbicide, was the cheapest and most cost-effective, while hand pulling was the most expensive and least cost-effective. This suggests that management cost is a key driver of the cost-effectiveness analysis for this particular species. All herbicides had available cost ranges but not hand pulling (Fig 12.1). Two pairs of actions had overlapping cost ranges making rank 1 and 2 as well as rank 2 and 3 for cost-effectiveness uncertain.

Because all actions achieve a declining population and actions target the same life stages, it could be expected that decisions could be made through cost considerations alone. Particularly, using cost as a proxy would be ideal when constructing demographic models is not feasible and externalities of the cost-effectiveness analysis are not affecting the decision-making process.


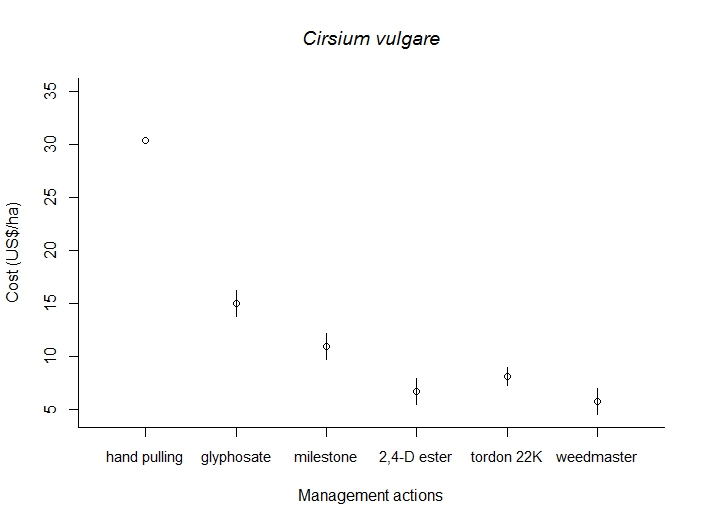


**Figure 12.1.** Cost and cost ranges (US$ per ha) for hand pulling and the five herbicides used to control *Cirsium vulgare* in pastures and along roadsides of Nebraska, USA. Circles represent mean cost estimates, and lines represent range in cost values for each action where data were available.

From the survey responses, the key decision-making factors for managing *Cirsium vulgare* in pastoral systems and roadsides in Nebraska were economic and biological considerations. None of the manager rankings matched cost-effectiveness or any of the proxies, yet all managers recommended Milestone as the first preference method for controlling *Cirsium vulgare* and hand pulling as the last preference method.

References

Bullock, J.M., B.C. Hill, & Silvertown, J. (1994). Demography of Cirsium Vulgare in a Grazing Experiment. *Journal of Ecology*, **82**, 101-111.

Doll, J. (2000). Bull, Plumeless, Musk and Canada Thistle: their biology and management in pastures. University of Wisconsin, Madison. <http://www.uwex.edu/ces/crops/uwforage/thistle.pdf>

Dow AgroSciences LLC. (2011). Biennial Thistle Management. Techline Newsletter. *Prairie and grassland Edition*, Spring 2011, 10-11. <http://weedwatchers.org/media/Document_26.pdf>

Deneke, Darrell, Moechnig, M., Vos, D., & Alms, J. (2012) Optimal Herbicide Application timing for Canada Thistle Control. South Dakota State University, Brookings. <http://techlinenews.com/articles/2013/1/25/optimal-herbicide-application-timing-for-canada-thistle-control>

Tenhumberg, B., S.M. Louda, J.O. Eckberg, & Takahashi, M. (2008). Monte Carlo analysis of parameter uncertainty in matrix models for the weed Cirsium vulgare. Journal of Applied Ecology, **45**, 438-447.

UNL. (2011). Guide to Weed Management in Nebraska with Insecticide and Fungicide Information: Weed Control by Noncrop Areas. University of Nebraska, Lincoln (UNL), NE. <http://nlcs1.nlc.state.ne.us/epubs/U2250/H001.0130-2011.pdf>
