# Supplementary material for: Prioritizing management actions for invasive populations using cost, efficacy, demography and expert opinion for 14 plant species world‐wide
Source: J Appl Ecol. 2016 Feb 22;53(2):305–16. doi: 10.1111/1365-2664.12592 (PMC4949517; doi:10.1111/1365-2664.12592)
Supplement: Supplementary file 13 — Appendix S13. Cytisus scoparius. [file JPE-53-305-s013.docx]

**Appendix S13**. ***Cytisus scoparius***

Fact sheet for management of low-density populations of *Cytisus scoparius* along the Shoalhaven River in New South Wales, Australia and invasive edge populations in prairies fields and Seattle city parks of Washington, USA.

Methods

We used existing matrices developed for *Cytisus scoparius* (common name, Scotch Broom) in New South Wales, Australia ([Stokes, Buckley & Sheppard 2006](#_ENREF_14)) and Washington, USA ([Parker 2000](#_ENREF_9)). The Australian matrix was parameterized for low-density (cover of <50%) populations of Scotch Broom along the Shoalhaven River in the southern tablelands of New South Wales ([Sheppard *et al.* 2002](#_ENREF_13); [Stokes, Buckley & Sheppard 2006](#_ENREF_14)). The Washington matrices were developed for populations of Scotch Broom at three city parks in Seattle and three prairie fields on Fort Lewis Military Base (now Joint Base Lewis-McChord) across the edge, intermediate, and centre stages of an invasion. We only used matrices for the edge stage of an invasion to due density independence assumption of matrix models ([Parker 2000](#_ENREF_9)).

For Washington populations, the matrix models partitioned the life cycle into three early stage classes - seed, seedling (first-year germinant), and juvenile (larger than biggest first year germinant to <5 mm diameter) - based on life history and four adult classes – small (<100 g), medium (100-400 g), large (400-900 g), and extra-large (>900 g) plants – based on biomass (Parker 2000). For the Australian population, the life history was also partitioned into seven similar classes based on plant height (Paynter, Downey & Sheppard 2003).

We contacted the Fort-Lewis Military Base (USA), Southern Rivers Catchment Management Authority (Australia), local councils and other agencies affiliated with the study sites for management data. Since the life stages of Scotch Broom were defined using height and age and height of Scotch Broom are correlated ([Paynter, Downey & Sheppard 2003](#_ENREF_10)), we were able to obtain life stage targets of control methods in the form of either height or age from managers. Cost estimates were converted to same units of measurement (cost per hectare) and US dollars (17 February 2012, www.oanda.com) in order to compare across species and sites. Biocontrol agents have been released in both Australia and United States with no success at significantly impacting populations. See Methods section of main text for more details on data analysis.

Results

Elasticity was able to completely discriminate between management actions for all sites, except the Australian population. However, management cost was the only proxy that perfectly aligned with cost-effectiveness for all sites. Weed wrench and pulling, prescribed fire, and cut stump were the most cost-effective for city parks, prairie fields, and the Australian population, respectively.

All management actions for the Australian population and only spot spraying for prairie fields were unable to achieve a declining population. However, assuming that the cost-lambda curve is linear, would it be possible to spend less on prescribed fire to population growth rate just below 1 or alternatively spend slightly more money on spot spraying (US$21.35 per ha instead of US$20.64 per ha) to achieve a declining population (Fig 13.1). Overall, the low-density population along the Shoalhaven River in Australia was the least cost-effective to manage, while the populations in prairie fields of Washington were the most cost-effective.


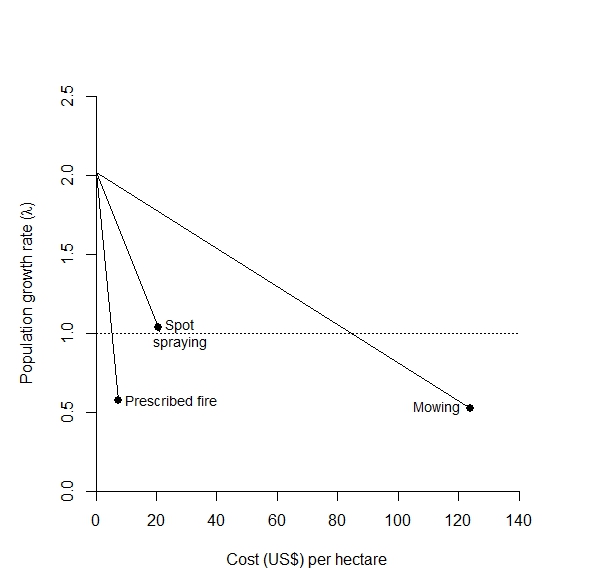


**Figure 13.1.** The assumed linear relationship between resulting population growth rate (λ) and cost of management actions used to control *Cytisus scoparius* in prairie fields of Washington, USA. The solid line represents the change the total reduction in population growth rate resulting from management, while the dotted line represents the additional cost required to achieve a declining population assuming a linear relationship.

From the surveys, the most common decision-making factors for managing populations of *Cytisus scoparius* management across the three sites were efficacy, demography, and environmental impacts. Across all sites, manager rankings did not align with cost-effectiveness or any proxies across all sites.

References

Parker, I.M. (2000). Invasion Dynamics of *Cytisus scoparius:* A matrix model approach. *Ecological Applications*, **10**, 726-743.

Paynter, Q., Downey, P.O., & Sheppard, A.W. (2003). Age Structure and growth of the woody legume weed *Cytisus scoparius* in native and exotic habitats: implications for control. *Journal of Applied Ecology*, **40**, 470-480.

Sheppard, A.W., Hodge, P., Paynter, Q., & Rees, M. (2002). Factors affecting invasion and persistence of broom *Cytisus scoparius* in Australia. *Journal of Applied Ecology*, **39**, 721-734.

Stokes, K.E., Buckley, Y.M., & Sheppard, A.W. (2006). A modeling approach to estimate the effect of exotic pollinators on exotic weed populations dynamics: bumblebees and broom in Australia. *Diversity and Distribution*, **12**, 593-600.
