# Supplementary material for: Prioritizing management actions for invasive populations using cost, efficacy, demography and expert opinion for 14 plant species world‐wide
Source: J Appl Ecol. 2016 Feb 22;53(2):305–16. doi: 10.1111/1365-2664.12592 (PMC4949517; doi:10.1111/1365-2664.12592)
Supplement: Supplementary file 14 — Appendix S14. Dipsacus sylvestris. [file JPE-53-305-s014.docx]

**Appendix S14.** ***Dipsacus sylvestris***

Fact sheet for management of *Dipsacus sylvestris* populations in Michigan, USA.

Methods

Werner and Caswell (1977) published stage-based matrix model for the biennial invasive herb, *Dipsacus sylvestris* (or common name Teasel), in eight fields within the Kalamazoo County in Michigan, USA (see Caswell 2001 for updated matrix). These small field plots differed significantly in vegetative structure from low to high composition of grass, litter, herbaceous dicots and woody dicots all in varying combinations. Although some individuals have been recorded to survive up to 5 years, the matrix model partitioned its biennial life history into seven life stages: seeds, dormant seeds (one year), dormant seeds (two years), small rosettes, medium rosettes, large rosettes, and flowering plants based off developmental, age and size characteristics (Werner & Caswell 1977). Managers were only able to provide management data for two common herbicides – Garlon 3A and 2,4-D Ester – used to control *Dipsacus sylvestris* and other invasive herbs in Michigan. See Methods section of main text for more details on data analysis.

Results

Both management actions received the same elasticity and efficacy value. Managers provided the same efficacy information, potentially due to lack of knowing the differences in management effects on target transition rates. Subsequently, management cost aligned with cost-effectiveness ranks. Garlon 3A was the cheapest and most cost-effective herbicide across all field sites, while both herbicides were able to theoretically achieve local extinction.

All managers contacted do not actively control populations of *Dipsacus sylvestris*, so management data was limited to two common herbicides. No managers were able to provide their opinion on which methods are preferential for managing *Dipsacus sylvestris* in Michigan, USA.

References

Caswell, H. (2001). Matrix Population Models: Construction, Analysis, and Interpretation. 2nd edition. Sinauer Associates Inc., Dusseldorf.

Werner, P.A. and Caswell, H. (1977). Population growth rates and age versus stage-distribution models for Teasel (*Dipsacus sylvestris* Huds.). *Ecology*, **58**, 1103-1111.
