# Supplementary material for: Prioritizing management actions for invasive populations using cost, efficacy, demography and expert opinion for 14 plant species world‐wide
Source: J Appl Ecol. 2016 Feb 22;53(2):305–16. doi: 10.1111/1365-2664.12592 (PMC4949517; doi:10.1111/1365-2664.12592)
Supplement: Supplementary file 15 — Appendix S15. Lespedeza cuneata. [file JPE-53-305-s015.docx]

**Appendix S15. *Lespedeza cuneata***

**.** Fact sheet for management of *Lespedeza cuneata* populations under mean herbivory conditions in Missouri, USA.

Methods

We used a matrix developed for populations of *Lespedeza cuneata* under mean herbivory conditions in Washington University’s Tyson Research Centre, Missouri. The matrix model partitioned the life history into six stage classes: cleistmogamous (self-pollinated) and chasmogamous (often insect-pollinated) seeds, and small (1 branch), medium (2-5 branches), large (6-10 branches), and extra-large (>10 branches) plants (Schutzenhofer, Valone & Knight 2009).

In Missouri, the main method of control for *Lespedeza cuneata* is chemical control. We accrued management data for our analyses from managers at the Missouri Department of Conservation as well as efficacy studies conducted by researchers at the University of Missouri. We then converted the herbicide costs to application rates used in these efficacy studies. We found six management actions, all herbicides, for controlling populations of *Lespedeza cuneata*: Cimarron, Chaparral, Surmont, PastureGard, Remedy Ultra, and Crossbow. See manuscript for more information on data analyses. See Methods section of main text for more details on data analysis.

Results

Management cost aligned the most with the cost-effectiveness out of all the management proxies. Elasticity, similar to *Cirsium vulgare*, was unable to discriminate between all actions used in our analysis. The efficacy analysis provided more discrimination than elasticity, yet did not align with cost-effectiveness. Management cost aligned the most with cost-effectiveness; this proxy could be used as a substitute for cost-effectiveness when no externalities of this analysis are of management concern. However, none of the actions used in our analysis were unable to theoretically reduce population growth rate to a declining rate.

Managers ranked these herbicides primarily on their effectiveness at controlling *Lespedeza cuneata* in Missouri. Consequently, their rankings aligned with the efficacy analysis the most out of all proxies. Since all actions used for *Lespedeza cuneata* are classified as chemical control, the reason for cost not influencing decision-making is potentially due to the low variation in cost (US$4.6-13.7 per ha) compared to when you have a variety of management actions under different categories, e.g. chemical vs. mechanical control.

References

Schutzenhofer, M. R., T. J. Valone, and T. M. Knight. (2009). Herbivory and population dynamics of invasive and native Lespedeza. *Oecologia*, **161**, 57-66.
