# Supplementary material for: Prioritizing management actions for invasive populations using cost, efficacy, demography and expert opinion for 14 plant species world‐wide
Source: J Appl Ecol. 2016 Feb 22;53(2):305–16. doi: 10.1111/1365-2664.12592 (PMC4949517; doi:10.1111/1365-2664.12592)
Supplement: Supplementary file 16 — Appendix S16. Parkinsonia aculeata. [file JPE-53-305-s016.docx]

**Appendix S16. *Parkinsonia aculeata***

Fact sheet for management of *Parkinsonia aculeata* in invaded areas of Western Australia and Northern Territory, Australia.

Methods

Using Pichancourt and van Klinken (2012), we developed matrices for populations of *Parkinsonia aculeata* in the semi-arid regions of Western Australia (WA) and the Northern Territory (NT). We aligned the climate district maps from the Bureau of Meteorology (BOM) with the *Parkinsonia aculeata* distribution map (see maps from Pichancourt & van Klinken 2012) to determine which climate districts of WA and NT are infested with *Parkinsonia aculeata*. We used the 2011 annual rainfall data from BOM to calculate the average total rainfall for each infested climate district. Using these data, we were able to calculate seed germination as well as the growth and survival of *Parkinsonia aculeata* needed to develop the matrices for each climate district (Pichancourt & van Klinken 2012).

Most management data for *Parkinsonia aculeata* were available from the literature at sites near Charters Towers in Queensland (McKenzie *et al.* 2004; Vitelli & Madigan 2004). We assumed that these estimates of costs and efficacy could be extrapolated inter-state to the invaded areas of Northern Territory and Western Australia used to parameterize these matrices. Basal bark application is one of the most commonly used methods for controlling infestation of *Parkinsonia aculeate*, and management data were available for sites used to parameterize these matrix models (L. Anderson., pers. comm., 10 February 2012; C. Rummery., pers. comm. 16 January 2012; N. Wilson., pers. 15 comm. 21 November 2011). In Pilbara, basal bark costs an average of US$562 per hectare in 2012 (cost range US$403-720 per ha) (L. Anderson, pers. comm., 10 February 2012), whereas basal bark had an estimated cost of AU$412 per ha in Queensland in 2001, which is equivalent to US$577.4 in 2012 (McKenzie *et al.* 2004). Given the large range in cost among actions (US$28-1354 per ha), we decided that these cost estimates are close that management data for other actions were used from the literature. All these cost estimates were converted to present value in 2012 (Reserve Bank of Australia; [www.rba.gov.au/calculator/](http://www.rba.gov.au/calculator/)) and the US dollar (17 February 2012, www.oanda.com) to compare across species and sites.

For our study, we used 10 management actions commonly used to control *Parkinsonia aculeata*: double chain, stick raking, Ellrott plough, blade plough, grubbing, soil application of Velpar and Grazon DS, basal bark application, aerial foliar application, and flame thrower. Three biocontrol agents have been released in invaded areas of Australia, yet no significant impacts on populations have been recorded. See Methods section of main text for more details on data analysis.

Results

No proxies aligned with cost-effectiveness ranks. Double chain pulling was the most cost-effective method, yet we found that aerial foliar application was the preferred action if the objective is to achieve a declining population. Seven out of 10 management actions could achieve a declining population of *Parkinsonia aculeata* in Australia. These results highlight the importance of considering management objectives when making recommendations since the most cost-effective action may differ based on whether you want to achieve a declining population or just any reduction in population growth rate.

Additionally, the cost-effectiveness analysis could be used to inform integrated management. For example, managers could use double chain pulling to reduce population growth rate to 1.16 then switch to aerial foliar application to reduce λ below 1 assuming that cost-lambda curve is linear (see Discussion section of manuscript for more details) (see Fig. 4 in manuscript). In this case, this integrated approach would be cheaper than just using aerial foliar application - the most cost-effective action at achieving a declining population - to control populations of *Parkinsonia aculeata*.

After contacting managers across Northern Territory and Western Australia, we found that basal bark application was the most commonly used and, in many cases, the only action used to control *Parkinsonia aculeata*. Only one manager provided ranks that did not align with any proxies or cost-effectiveness; their reasoning was cost, demographic targets, environmental impacts, time-consumption, and viability (e.g. difficulty of use, legal status). These results suggest that decision-making factors go beyond the parameters of the cost-effectiveness, and that other externalities contribute towards which actions are used for controlling invasive populations.

References

McKenzie, J. R., M. J. Pattison, K. E. Steele, S. D. Campbell, & J. S. Vitelli. (2004). Controlling dense infestations of parkinsonia (Parkinsonia aculeata L.). *Fourteenth Australian Weeds Conference*, 176-178.

Pichancourt, J.B. & R. D. van Klinken. (2012). Phenotypic plasticity influences the size, shape and dynamics of the geographic distribution of an invasive plant. *PLOS ONE*, **7**, e32323.

Vitelli, J. S. & B. A. Madigan. (2004). Evaluation of a hand-held burner for the control of woody weeds by flaming. *Australian Journal of Experimental Agriculture*, **44**, 75-81.
