# Supplementary material for: Prioritizing management actions for invasive populations using cost, efficacy, demography and expert opinion for 14 plant species world‐wide
Source: J Appl Ecol. 2016 Feb 22;53(2):305–16. doi: 10.1111/1365-2664.12592 (PMC4949517; doi:10.1111/1365-2664.12592)
Supplement: Supplementary file 17 — Appendix S17. Persicaria perfoliata. [file JPE-53-305-s017.docx]

**Appendix S17. *Persicaria perfoliata***

Fact sheet for management of *Persicaria perfoliata* populations in nature reserves in Pennsylvania and Delaware, USA.

Methods

Hyatt and Araki (2006) developed stage-structured periodic matrix models for annual invasive vine, *Persicaria perfoliata* (previously, *Polygonum perfoliatum*), for two populations within three nature reserves of Pennsylvania, USA across 1999 and 2000. The three nature reserves were Ridley Creek State Park, Tyler Arboretum and Burrow Runs Preserve. This periodic matrix partitioned the life history into five stages: seeds, small seedlings, medium seedlings, large seedlings and plants based on size and developmental characteristics to project changes to these stages within the populations across the seasons.

For *Persicaria perfoliata*, management actions are used at different periods during the growing season, e.g. Pendulum aquacap is an early season (March-April) herbicide while Garlon 3A is a mid-late season (May-July) herbicide. We used the appropriate periodic matrix for each action (see S1 and S3.1 for details). Managers were able to provide management data for hand pulling and three herbicides: Pendulum aquacap, Plateau and Oust, and Garlon 3A. A biocontrol agent, *Rhinoncomimus latipes*, has been released in Pennsylvania and neighbouring states with recorded impacts on populations (Hough-Goldstein, Lake, & Reardon 2012); however, we had to exclude biocontrol actions from our study due to differences in cost structure and time-scale effects from local management actions. See Methods section of main text for more details on data analysis.

Results

We found that the cost-effectiveness of management actions differed across three nature reserves (f = 14.68, d.f. = 2, *P* < 0.0001) with populations at Ridley Creek State Park being the most cost-effective to manage (0.015 $USD^-1^ ha^-1^ ± 0.002) and Tyler Arboretum the least cost-effective (0.003 $USD^-1^ ha^-1^ ± 0.0008). No other metrics had a significant effect of site. Surprisingly, all actions received the same elasticity despite targeting different transition rates in different periodic matrices. Efficacy analysis provided more discrimination between actions, yet only management cost seemed to align with cost-effectiveness whereby 88.5% of actions across all sites received same rank for cost and cost-effectiveness. No other management proxy aligned with cost-effectiveness ranks. Across all six study sites, all management actions were able to achieve a declining population of *Persicaria perfoliata* and therefore, cost could be used as a substitute for the cost-effectiveness analysis if only cost, efficacy and demography are the factors influencing decision-making.

For these four methods, managers considered effectiveness and environmental impacts as the key drivers for ranking these actions. Two managers said they were able to reliably rank these four methods based on individual merit, yet none of these rankings aligned with our proxies (elasticity, efficacy, or cost) or cost-effectiveness. Our analysis found that the most cost-effective action was hand pulling, while Pendulum aquacap was often ranked last according to cost-effectiveness. Both managers ranked the herbicide, Pendulum aquacap, as the preferred action to control *Persicaria perfoliata* populations because it has little off-target impacts on the surrounding environment. Our findings suggest that we need to create a better multi-criteria analysis that incorporates other management concerns, such as off-target impacts.

References

Hough-Goldstein, J., Lake, & Reardon, R. (2012). Status of an ongoing biological control program for the invasive vine, *Persicaria perfoliata*, in eastern North America. *BioControl*, **57**, 181-189.

Hyatt, L. and Araki, S. (2006). Comparative population dynamics of an invading species in its native and novel ranges. *Biological Invasions*, **8**, 261-275.
