# Supplementary material for: Prioritizing management actions for invasive populations using cost, efficacy, demography and expert opinion for 14 plant species world‐wide
Source: J Appl Ecol. 2016 Feb 22;53(2):305–16. doi: 10.1111/1365-2664.12592 (PMC4949517; doi:10.1111/1365-2664.12592)
Supplement: Supplementary file 18 — Appendix S18. Pinus nigra. [file JPE-53-305-s018.docx]

**Appendix S18. *Pinus nigra***

Fact sheet for management of low-density populations of *Pinus nigra* on Mt Barker, New Zealand.

Methods

We used a matrix developed for low-density populations of Corsican Pine, *Pinus nigra*, on Mount Barker in the south island of New Zealand (Caplat, Nathan & Buckley 2012). This matrix is an improved version of the matrix published in Buckley et al. 2005. The matrix is an age-based model with four classes: seedlings (0-1 year olds), juveniles (1-10 years), sub-adult (10-15 years) and adult plants (15+ years).

The Wilding Control Program from the New Zealand Wilding Conifer Management Group (NZWCMG) listed all the possible control methods, their costs, and the life stages these control methods target according to the height and diameter at breast height (DBH) of Pines (Ledgard 2009). In order to assign the height and diameter-based estimates of management cost to the matrix transitions, we estimated the relationship between diameter and age and between height and age using linear regression. We found that height is a better indicator of age (R^2^= 0.92) than diameter at breast height (R^2^=0.87) (Fig 18.1).


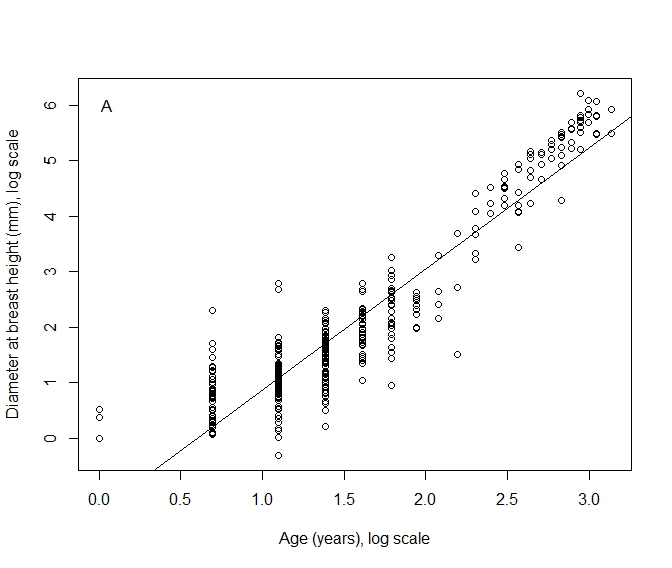

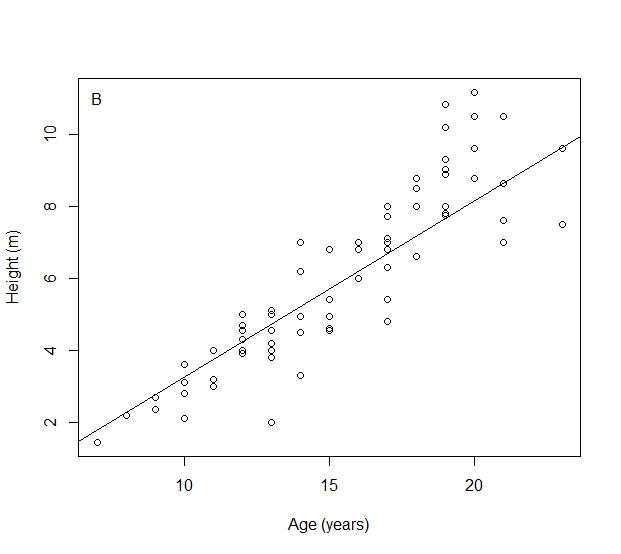


**Figure 18.1.** The relationship between age and (A) diameter at breast height (F=2652, df=383, p<0.001) and (B) height above 1.37 meters, the height for measuring diameter at breast height (F=4154, df=381, p<0.001) (Buckley unpublished data).

We converted all costs from the Wilding Control Program from 2008 to present value in 2012 (www.rbnz.govt.nz). We contacted managers and researchers at Scion Research, NZWCMG, New Zealand Department of Conservation, Pan Pac Forest Products, and AG Designs and asked them questions about the cost and efficiency data that was missing from the Wilding Control Program. See methods section of main text for more details on data analysis.

Results

Management actions received one of four elasticity values; this may be reflective of the limitations of life history partitioning of matrix models. Restricting the life history partitioning to four stages may have resulted in the poor distinction between how population growth rate changes with small changes to life stages targeted by management. Efficacy analysis was able to more clearly differentiate the effects of management on population growth rate compared to elasticity analysis.

We found that none of the management proxies aligned with cost-effectiveness ranks suggesting none of these parameters drive the outcome of the economic sensitivity analysis for this species. Out of the three management proxies, management cost seemed to have the least confliction with cost-effectiveness. Five out of 13 management actions had cost range data, and actions ranked 2 & 3 and 5 & 8 had some overlap (Fig 18.2). These overlaps suggest that coarser estimates of costs are required to make proper recommendation for management; however, hand pulling was the most cost-effective method by almost 7 fold compared to the second cost-effective method. None of these actions could individually achieve a declining population for *Pinus nigra*.


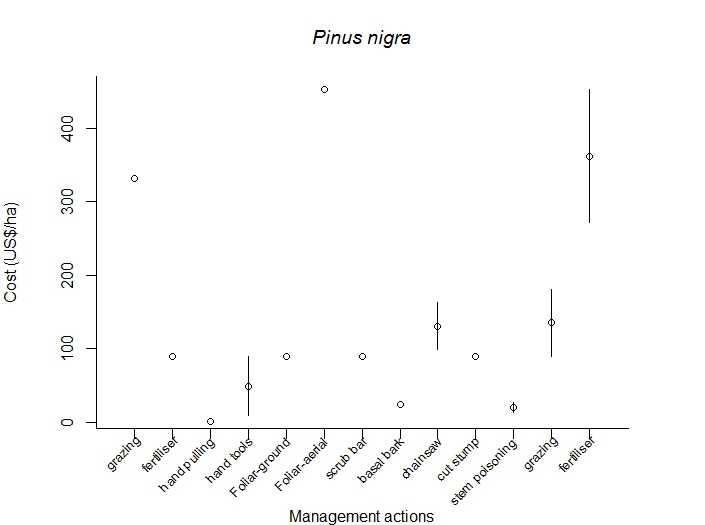


**Figure 18.2.** Cost point and ranges (US$ per ha) for 13 management actions used to control *Pinus nigra* in New Zealand. Circles represent range in cost values, and lines represent range in cost values for actions where data were available. Hand pulling still remained the cheapest method when comparing with minimum cost values of other actions.

We received a response from one manager that could provide feedback on the individual merits of only five of the 13 actions for controlling populations of *Pinus nigra* on Mt Barker, New Zealand (see Buckley *et al.* 2005; Caplat, Nathan & Buckley 2012 for details on population). Their key considerations when ranking actions were demographic targets, effectiveness, cost, time consumption, and viability concerning site and population characteristics. Although actions could be ranked based on their impact on the population, the manager said that not one method would be entirely effective at controlling the whole population because of the developmental complexity means that one method is not the most suitable or effective at targeting the full range of life stages.

References

Buckley, Y., E. Brockerhoff, L. Langer, N. Ledgard, H. North, & M. Rees. (2005). Slowing down a pine invasion despite uncertainty in demography and dispersal. *Journal of Applied Ecology*, **42**, 1020-1030.

Caplat, P., R. Nathan, & Y. Buckley. (2012). Seed terminal velocity, wind turbulence and demography drive the spread of an invasive tree in an analytical model. *Ecology*, **93**, 368-377.

Ledgard, N.J. (2009). Wilding Control: Guidelines for the Control of Wilding Conifers. New Zealand Forest Research Institute (SCION), Fendalton, Christchurh.
