# Supplementary material for: Prioritizing management actions for invasive populations using cost, efficacy, demography and expert opinion for 14 plant species world‐wide
Source: J Appl Ecol. 2016 Feb 22;53(2):305–16. doi: 10.1111/1365-2664.12592 (PMC4949517; doi:10.1111/1365-2664.12592)
Supplement: Supplementary file 19 — Appendix S19. Prunus serotina. [file JPE-53-305-s019.docx]

**Appendix S19. *Prunus serotina***

Fact sheet for management of *Prunus serotina* populations under light conditions in Compiègne Forest, northern France.

Methods

Sebert-Cuvillier *et al.* (2007) developed a stage-based matrix model for populations of *Prunus serotina* in light conditions of Compiègne forest, northern France. This model partitions the life history into seven discrete stages based on developmental stage, size and seed dormancy: seeds-1 (germinate first spring), seeds-2 (germinate second spring), seedling, sapling-1 to sapling-7 (based on height) and adult (Sebert-Cuvillier *et al.* 2007).

We obtained site and species-specific management data for our cost-effectiveness analysis by contacting managers from Compiègne forest. Managers were able to provide management data for seven actions currently used to control *Prunus serotina* within the forest. These actions are felling, cutting with occasional debarking, ring barking, glyphosate, triclopyr, cut stump and hand pulling. Sheep grazing and fungal herbicide (*Chondrostereum purpureum*) are relatively new methods that are currently under investigation, so they were not included within our study. See Methods section of main text for more details on data analysis.

Results

Efficacy analysis provided slightly more distinction between management actions than the elasticity, yet cost was the only proxy that aligned with cost-effectiveness ranks. Ring barking was the cheapest and most cost-effective action for managing *Prunus serotina* in Compiègne forest. However, all seven actions had cost ranges, and cost ranges of these actions do overlap (Fig 19.1). Actions ranked 1 to 3 in cost-effectiveness overlap, while actions ranked 4 to 6 also overlap. This overlapping cost ranges suggest that ring barking may not remain the most cost-effective action under different cost scenarios. All management actions were able to theoretically achieve local extinction.


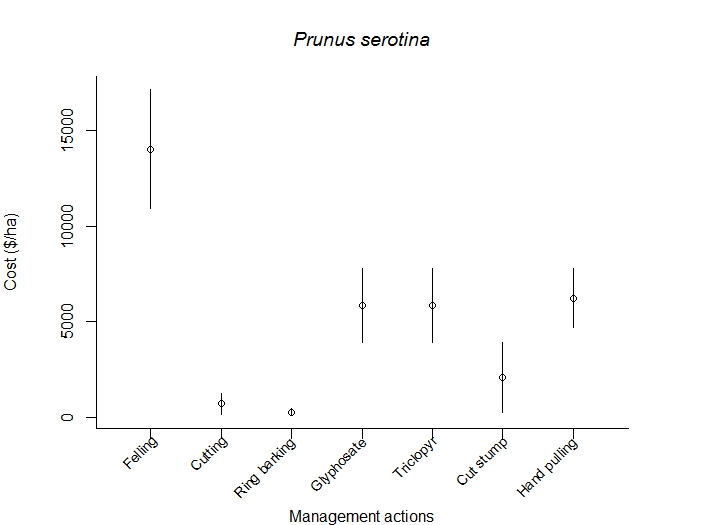


**Figure 19.1.** Cost points and ranges (US$ per ha) for the seven management actions used to control *Prunus serotina* in Compiègne forest, France. Circles represent mean cost estimates, and lines represent range in cost values for each action where data were available.

We received one manager response to our survey on management *Prunus serotina* in Compiègne Forest; these manager rankings did not align with any of the proxies. Ring barking was ranked first by the manager for controlling *Prunus serotina* similar to cost-effectiveness. However, only three out of the six actions received the same ranks for both manager rankings and cost-effectiveness. The manager did consider cost and efficacy when ranking these actions, but they also considered environmental impacts that may explain the differences in some ranks compared to cost-effectiveness.

References

Sebert-Cuvillier, E., Paccaut, F., Chabrerie, O., Endels, P., Goubet, O., & Decocq, G. (2007). Local population dynamics of an invasive tree species with a complex life-history cycle: A stochastic matrix model. *Ecological modelling*, **201**, 127-143.
