# Supplementary material for: Prioritizing management actions for invasive populations using cost, efficacy, demography and expert opinion for 14 plant species world‐wide
Source: J Appl Ecol. 2016 Feb 22;53(2):305–16. doi: 10.1111/1365-2664.12592 (PMC4949517; doi:10.1111/1365-2664.12592)
Supplement: Supplementary file 20 — Appendix S20. Rubus armeniacus. [file JPE-53-305-s020.docx]

**Appendix S20.** ***Rubus armeniacus***

Fact sheet for management of low-density populations of *Rubus armeniacus* in forested areas of Oregon, USA.

Methods

We used the existing matrices developed for low-density populations of *Rubus armeniacus* in Oregon ([Lambrecht-McDowell & Radosevich 2005](#_ENREF_1)). These matrices partitioned the life history of Rubus *armeniacus* into four developmental stages: seedlings, canes (s) developed from seedlings, reproductive canes, and canes (v) that developed from tip-rooting or rhizome sprouting.

We searched the literature (e.g. Web of Science) and grey-literature (e.g. government websites, google, google scholar) for information on cost and efficiency using search terms such as management, cost, *Rubus armeniacus,* Himalayan Blackberry, Oregon. We found cost data for different management actions in a Nature Conservancy publication on controlling *Rubus armeniacus* in the Pacific Northwest ([Soll 2004](#_ENREF_2)). All cost data from the Nature Conservancy publication were converted from the first quarter of 2004 to present value in quarter 4, 2011 (BLS 2015). We then contacted researchers and managers at the U.S. Forest Service and Oregon Department of Agriculture and asked questions about management actions, life stages targeted by actions, and management efficacy. See Methods section of main text for more details on data analysis.

Results

Efficacy ranks did not align with elasticity for management of *Rubus armeniacus* indicating the importance of considering the management effects on vital rates targeted. Only cost aligned with cost-effectiveness out of all the proxies. All management strategies were able to achieve a declining population of *Rubus armeniacus*. However, the applicability of our study to *Rubus armeniacus* is far more complex than any of the other species in our study due to its ability to reproduce both sexually and clonally making it difficult to properly estimate the effects of management on life stages.

All three managers provided the same ranks for *Rubus armeniacus* management used in our study with their reasons being cost, effectiveness and time consumption. Despite considering the same parameters to rank actions as the cost-effectiveness analysis, these rankings did not align with cost-effectiveness. Coincidentally, these ranking aligned with elasticity analysis, despite only one manager accounting for demographic considerations when ranking actions. Since managers considered the same parameters as the cost-effectiveness, this mismatch in ranks could suggest the need for a cost-effectiveness analysis to properly assess and weigh management actions, or the need for more courser estimates of management efficacy. The most cost-effective action was the use of a Weedeater, while managers unanimously ranked spot spraying as the best action for controlling *Rubus armeniacus.*

References

Bureau of labor statistics. (2015) CPI Inflation calculator. Division of Consumer Prices and Price Indexes, Washington DC. Available from: <http://www.bls.gov/data/inflation_calculator.htm>

Lambrecht-McDowell, S. and S. Radosevich. (2005). Population demographics and trade-offs to reproduction of an invasive and noninvasive species of Rubus. *Biological Invasions*, **7**, 281-295.

Soll, J. (2004). Controlling Himalayan Blackberry (Rubus armeniacus [R. discolour, R. procerus]) in the Pacific Northwest. *The Nature Conservancy*, <http://www.invasive.org/gist/moredocs/rubarm01.pdf>
